# Endonuclease Specificity and Sequence Dependence of Type IIS Restriction Enzymes

**DOI:** 10.1371/journal.pone.0117059

**Published:** 2015-01-28

**Authors:** Sverker Lundin, Anders Jemt, Finn Terje-Hegge, Napoleon Foam, Erik Pettersson, Max Käller, Valtteri Wirta, Preben Lexow, Joakim Lundeberg

**Affiliations:** 1 Science for Life Laboratory, KTH, Gene Technology, Solna, 171 65, Sweden; 2 LingVitae AS, Husøysund, 3132, Norway; The Scripps Research Institute, UNITED STATES

## Abstract

Restriction enzymes that recognize specific sequences but cleave unknown sequence outside the recognition site are extensively utilized tools in molecular biology. Despite this, systematic functional categorization of cleavage performance has largely been lacking. We established a simple and automatable model system to assay cleavage distance variation (termed slippage) and the sequence dependence thereof. We coupled this to massively parallel sequencing in order to provide sensitive and accurate measurement. With this system 14 enzymes were assayed (AcuI, BbvI, BpmI, BpuEI, BseRI, BsgI, Eco57I, Eco57MI, EcoP15I, FauI, FokI, GsuI, MmeI and SmuI). We report significant variation of slippage ranging from 1–54%, variations in sequence context dependence, as well as variation between isoschizomers. We believe this largely overlooked property of enzymes with shifted cleavage would benefit from further large scale classification and engineering efforts seeking to improve performance. The gained insights of in-vitro performance may also aid the in-vivo understanding of these enzymes.

## INTRODUCTION

Restriction endonucleases (REs) with shifted cleavage site relative to the site of recognition have been extensively utilized for nucleic acid analysis, e.g. for gene expression analysis [1–5] and massively parallel sequencing [6–8]. The shifted cleavage property enables manipulation of unknown nucleotide compositions enabling analysis of novel sequences. The primary group of enzymes with this property are the Type IIS REs with now over 417 enzymes in the restriction enzyme database (REBASE, http://rebase.neb.com, 8 October 2014) [9]. There have been reports of varying cleavage shifts (unspecific cleavage) for some enzymes in conjunction with developed protocols for sequencing, for example MmeI [6], AcuI and EcoP15I [8] in addition to early reports including MnlI [10–13], BcefI [14], BceSI [15] and HphI [16]. Sequence context dependency has been implicated for the cleavage by HphI [16]. In REBASE 45 enzymes are listed as having variable cuts (http://rebase.neb.com/cgi-bin/varcutlist, 8 October 2014) of which 37 are Type IIS REs. Details of to which extent this variation occurs, or correlations to sequence composition is not easily obtained or even available. Cleavage variation can be viewed as an analogue to star activity, i.e. the relaxation of recognition and subsequent unspecific cleavage of sequences other than the cognate recognition sequence [17, 18]. Star activity is a widely known behaviour of REs, and is influenced by certain buffer conditions, such as Mn^2+^ ions, pH and glycerol [17, 18]. REs are essential tools for molecular biology and efforts have recently begun to systematically classify and subsequently improve of this unspecific reactivity [18, 19]. Although Type IIS REs have been extensively studied and utilized, contrary to star activity, little has been reported on the variation in cleavage site distance and the sequence dependence of this variation, which also greatly influence the usefulness of this type of REs as in vitro reagents. Unlike most Type II REs, Type IIS REs are constituted by dual domains, one domain responsible for sequence recognition, and one domain responsible for endonuclease activity [20]. This activity separation necessitates separate studies to classify the endonuclease specificities. The recognition sequence specificity, and often also the RE’s star activity, is supplied by commercial manufacturers, whereas the sequence dependence and potential variance of the endonuclease activity is not. In the cases where the phenomenon has been reported in literature, the information is not easily accessible, despite its relevance in molecular biology protocol development. Motivated by this lack of information, a model system was devised to characterize the endonuclease activity of various Type IIS REs.

In this first systematic in-depth characterization of Type IIS endonuclease behaviour we report on a broad range of variation and sequence dependence. Thirteen Type IIS enzymes were selected, and one Type III enzyme, all of them demonstrating separated recognition and endonuclease activity (shifted cleavage). Primarily enzymes with relatively long distance shifts (16 bp was the most common distance) were assayed, since longer distances often provide in-vitro advantages when developing methods. Three pairs of isoschizomers, sharing the same recognition sequence, were included to investigate potential differences in endonuclease activity. A workflow was established for studying length specificity of shifted cleavage as well as sequence dependence, to assay the potentially varying distance shifts and the correlation to DNA sequence composition. The term slippage is used to describe the enzymes’ propensity to slip 1 or 2 bp away from the preferred site of cleavage. The focus was primarily technical, aiming to provide knowledge for further protocol development in nucleic acid in vitro manipulation, but we also expect the results to aid the general understanding of these enzymes, e.g. cleaving mechanisms, functionality and evolution, by characterizing the in-vitro differences.

## MATERIAL AND METHODS

**Figure 1:**
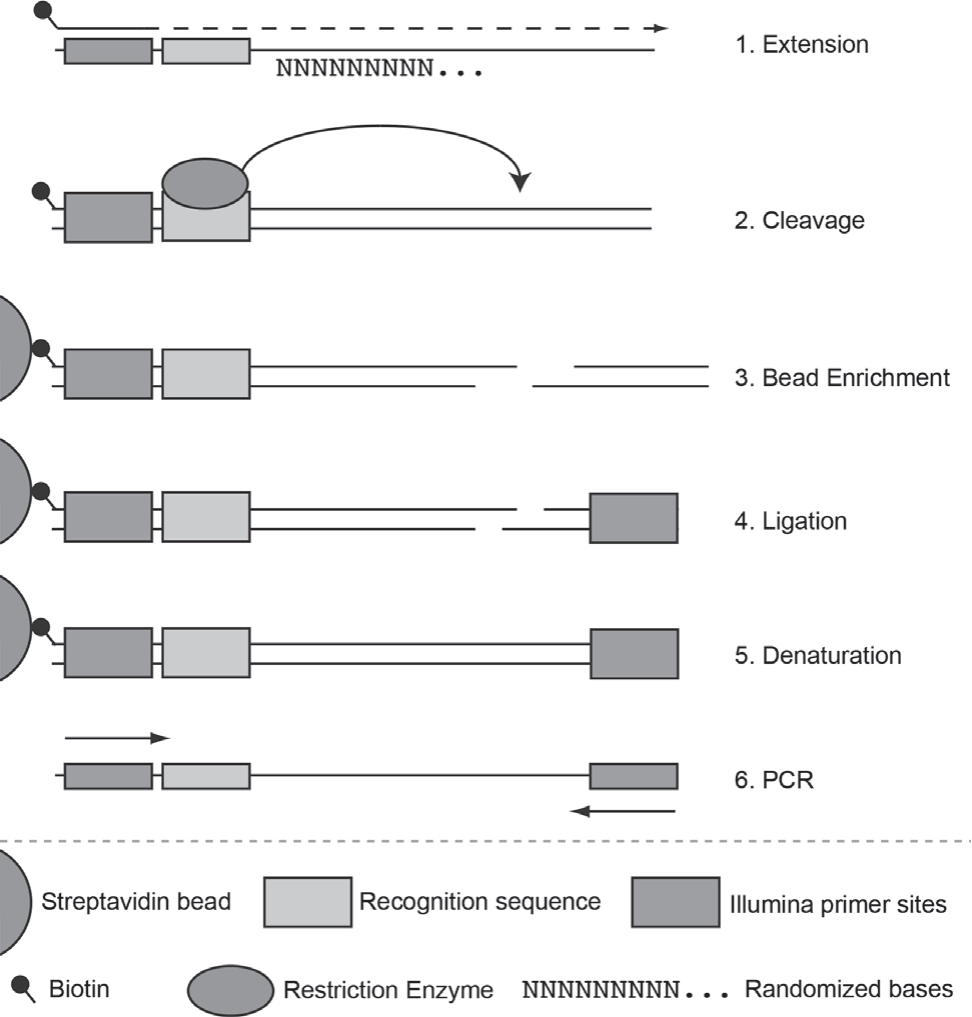
Schematic representation of the model system. The library preparation consists of six steps, beginning by extending randomized synthesized oligonucleotides (1), digesting the extended constructs (2), enriching the constructs and removing cleaved products (3), ligating sequencing adapters to the cleaved fragments (4), denaturing single stranded DNA from beads (5), and amplifying with PCR for sequencing (6).

A general overview of the method workflow is be depicted in Fig. 1, and a detailed description of the model system is included in (S1 Method). In short, the model system consists of synthesized oligonucleotides containing an Illumina primer site and an enzyme specific recognition site, downstream of which there are a series of randomized nucleotides spanning the expected cleavage distance. An infill reaction generates the templates, which are subsequently digested with a restriction endonuclease that cleaves into the randomized sequence. The digestion products are ligated to adapters that have overhangs with degenerate bases of matching lengths, a control sequence and an Illumina primer site. These constructs are sequenced and the distances between the recognition sites and the control sequences are analysed.

### Substrate preparation

Four 100 μl infill reactions were set up per substrate using 100 pmol template oligonucleotides (T1-T15 in S1 Method) and 200 pmol biotinylated infill primer (P1 in S1 Method) in 1x Klenow buffer (Thermo Scientific) supplemented to 200 μM dNTPs (Invitrogen). Hybridization was performed by heating to 95°C for 5 minutes, followed by cooling to 25°C. The reactions were then supplemented with 10 U Klenow fragment (Thermo Scientific) and incubated at 37°C for 30 min. Klenow fragment was heat inactivated at 65°C for 20 min. Each reaction was purified and concentrated to 40 μl using Qiaquick columns (Qiagen) and eluting in nuclease free water. Final concentrations were determined by Nanodrop (Thermo Scientific). Each pair of adapter oligonucleotide (A1–A4 in S1 Method) were annealed in 1x PNK buffer (NEB) by incubating 20 μM of each oligonucleotide at 94°C for 3 minutes and slowly ramping down to 25°C.

### Preparing the library

Each prepared substrate was treated with corresponding RE and incubated for 1 hour as described in Table 1, followed by heat inactivation. Dynal M-280 streptavidin beads (Invitrogen), 100 μl/sample were washed in 2x bind and wash buffer (2x BW: 10 mM Tris, 1 mM EDTA, 2 M NaCl), added to each reaction and incubated at room temperature (RT) for 30 min. The beads were washed 3 times in 1x TE and resuspended in 100 μl 1x BW. Half of the beads were resuspended in 22 μl 1x T4 DNA Ligase buffer (Thermo Scientific), and supplemented with 20 pmol adapter corresponding to the product formed (A1–A4 in S1 Method) and 5 U T4 DNA ligase (Thermo Scientific), incubated 1 hour at RT with agitation. The supernatant was removed and beads were washed. ssDNA was extracted by adding 12 μl 0.15 M NaOH for 5 min at RT with agitation. The supernatant was removed and neutralized with 6 μl 0.3 M HCl and 3 μl 10x ABL (100 mM Tris, 1 mM MgCl_2_). Ligation products were diluted 20×–500× as input to PCR. Four 50 μl PCR reactions per sample were set up with 1x HotStarTaq PCR Buffer (Qiagen), 200 nM dNTPs (Qiagen), 50 nM of primer (P2 and P3 in S1 Method), and 2.5 U HotStarTaq DNA Polymerase (Qiagen). The PCR was run at 95°C 15 min, [94°C 1 min, 55.5°C 1 min, 72°C 1 min] × 22 cycles, 72°C 10 min, 4°C hold. The four reactions of each sample were pooled and purified using MinElute spin columns (Qiagen) to 20 μl EB (Qiagen). 60 ng per sample were pooled and concentrated using a MinElute column to 13 μl, which was used for sequencing.

**Table 1:** Reaction conditions for each enzyme used in the study.

**Enzyme**	**Supplier**	**Temperature *(°C)***	**Buffer**	**SAM (μM)**	**Enzyme amount *(U)***	**Substrate amount *(pmol)***	**Reaction volume *(μl)***	**Heat inactivation *(°C/min)***
AcuI	N	37	4	40	40	20	50	65/20
BbvI	N	37	4	-	40	20	50	65/20
BpmI*	N	37	2	-	40	20	50	65/20
BpuEI	N	37	3	80	40	20	50	65/20
BseRI*	N	37	4	-	40	20	50	65/20
BsgI	N	37	4	80	10	10	10	65/20
Eco57I	T	37	G	10	40	20	50	65/20
Eco57MI	T	37	B	10	40	20	50	65/20
EcoP15I*,**	N	37	3	-	40	20	50	65/20
FauI	N	55	4	-	40	20	50	65/20
FokI	N	37	4	-	40	20	50	65/20
GsuI	T	30	B	-	10	5	10	65/20
MmeI	N	37	4	50	40	20	50	80/20
SmuI	T	37	Tango	-	40	20	50	65/20

* supplemented with BSA, 100 μg/ml

** supplemented with ATP, 1 mM

N = New England Biolabs

T = Thermo Scientific

SAM = S-adenosylmethionine

### Sequencing

The pooled prepared samples were sent for sequencing to GATC Biotech AG Next Gen Lab (Germany) and sequenced using an Illumina Genome Analyzer II, 1×76 bp obtaining 11.8 million reads. The raw sequencing reads are available at the NCBI Sequence Read Archive under accession SRS627913.

### Analysing the data

The sequence files were processed with custom made Python scripts. First, for each read in the raw data, the first six bases were matched to the indices on the adapters used for ligation. A hamming distance of two or less to the synthesized adapter was considered a match. A cleavage distance search was initially conducted on the entire reads, where perfect matches to 10 bp immediately before the randomized region of the designed constructs (digestion products in S1 Method) was used to determine cleavage distance. As the primary analysis the recognition sequence of each RE was matched in descending order from the enzyme with longest cleavage distance only matching at the preferred distance of cleavage for each RE +/− 2 bp. For each enzyme with a perfect match, at each distance (−2, −1, 0, +1, +2 bp), a sequence logo (Weblogo 3.4, [21] was made from all the unique sequences classified to that distance. Non-unique sequences were used for FauI and SmuI, as the shorter cleavage distance of these RE (6 bp for non-slipp cleavage) limited the available number of variants. To assay the impact of sequence quality, a very stringent filtering step where all reads were trimmed to 45 bp and any read containing a base with less then Q20 was discarded. The filtered file was then analysed in the same way as the non-filtered.

## RESULTS

The enzymes included in the model system (as outlined in Fig. 1) of this study were AcuI, BbvI, BpmI, BpuEI, BseRI, BsgI, Eco57I, Eco57MI, EcoP15I, FauI, FokI, GsuI, MmeI and SmuI. Several of the enzymes included are isoschizomers: AcuI and Eco57I (CTGAAG), BpmI and GsuI (CTGGAG), and FauI and SmuI (CCCGC). Eco57MI is an engineered version of Eco57I that recognize (CTGRAG), and both types of recognition sequences were included in our analysis. The number of sequence reads obtained were 11,815,749, of those 11,729,811 (99.3%) adapter tags were classified to a type of ligation adapter, and of those 9,684,767 (82.0%) sequences were classified as belonging to a particular recognition sequence and used for the analysis. In general, the experiment showed low background and specific matches for the enzymes (S1 Fig.).

### Slippage

**Figure 2:**
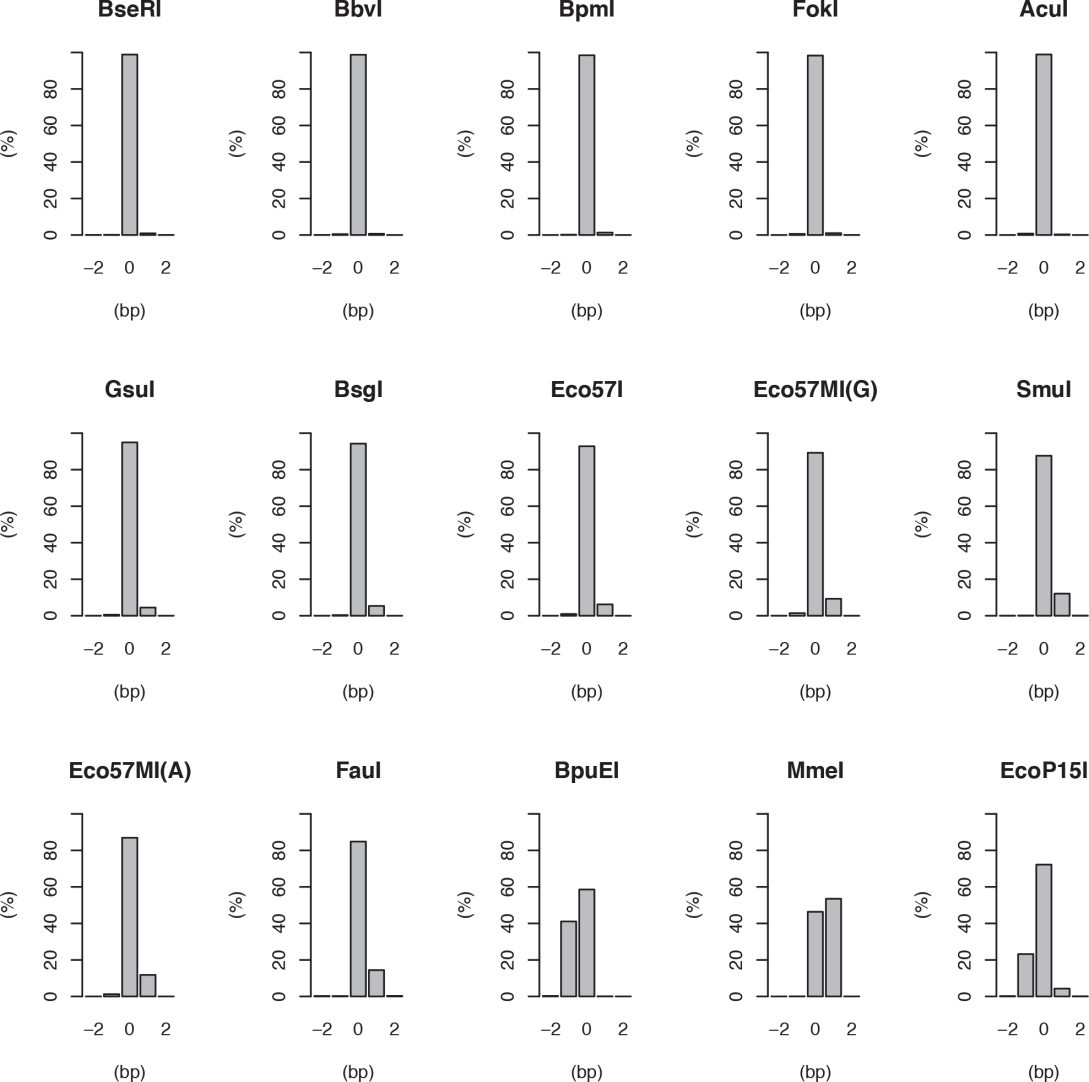
Slippage distributions for the Type IIS enzymes tested. Bar plots showing the percentage of reads obtained at the expected distance (0) and +/− 2 bp away for different Type IIS enzymes. The enzymes display a wide range of length specificity but usually not more than 1 bp away from the preferred distance.

For the set of enzymes studied the RE target templates digested by 1 or 2 bp off the defined distance varied from 1–54% (average 13.1%, median 7%) (Table 2). For this set of enzymes, roughly three classes of slippage frequency was observed, defined as low slippage (0–2%), intermediate slippage (2–15%), and high slippage (>15%). Low slippage enzymes were BseRI, AcuI, BbvI, BpmI and FokI, intermediate slippage enzymes were GsuI, BsgI, Eco57I, Eco57MI, SmuI and FauI, and high slippage enzymes were BpuEI, MmeI and EcoP15I (Fig. 2, Table 2). The enzyme least prone to slip was BseRI with 1.1% products +/− 2 bp of its defined distance, where +1 was the most common slippage product (0.9%). The enzyme most prone to slip was MmeI with a total slippage of 54% where +1 was almost the only product (Table 2, S1 Table). The second enzyme most prone to slip was BpuEI with 41% of the product formed missing one base in length. EcoP15I, the only Type III enzyme in this study, slipped in 26% of the cases. For Eco57MI, a modified version of Eco57I to recognize CTGRAG, the alteration between A to G slightly influenced the enzyme’s slippage, and Eco57MI displayed a slight increase in slippage as compared to the original enzyme (Eco57I). The detected slippage for Eco57I was 7%, and 11% for CTGAAG vs. 13% for CTGGAG (Table 2). The isoschizomers also varied. AcuI displayed 1.1% slippage and Eco57I 7.2%, BpmI 1.5% and GsuI 5.1%, and SmuI 12.4% and FauI 15.2%. Slipping is clearly not a random event of +/−1 base, but different enzymes show differing propensity for a particular direction. We found that a 2 bp variation is very rare. FauI showed the highest general tendency to slip +2 bp, at 0.31% and BpuEI had the highest tendency for −2 at 0.26%.

**Table 2:** Distribution of total slippage detected of the restriction enzymes.

**Enzyme**	**Expected distance (bp)**	Total slippage*	**Isoschizomer**	**Organism**
BseRI	10	1.1%	-	*Bacillus species R*
AcuI	16	1.1%	Eco57I	*Acinetobacter calcoaceticus*
BbvI	12	1.3%	-	*Bacillus brevis*
BpmI	16	1.5%	GsuI	*Bacillus pumilus*
FokI	13	1.7%	-	*Flavobacterium okeanokoites*
GsuI	16	5.1%	BpmI	*Gluconobacter suboxydans H-15T*
BsgI	16	5.7%	-	*Bacillus spaericus GC subgroup*
Eco57I	16	7.2%	AcuI	*Escherichia coli RFL57*
Eco57MI(G)	16	10.7%	-	*Escherichia coli RFL57M*
SmuI	6	12.4%	FauI	*Sphingobacterium multivorum RFL21*
Eco57MI(A)	16	13.1%	-	*Escherichia coli RFL57M*
FauI	6	15.2%	SmuI	*Flavobacterium aquatile*
EcoP15I	27	25.8%	-	*Escherichia coli P15*
BpuEI	16	41.4%	-	*Bacillus pumilus 2187a*
MmeI	20	53.6%	-	*Methylophilus methylotrophus*

*Slippage is defined as a cleaving 1 or 2 bases up- or downstream of the expected site of cleavage.

### Sequence dependence

**Figure 3:**
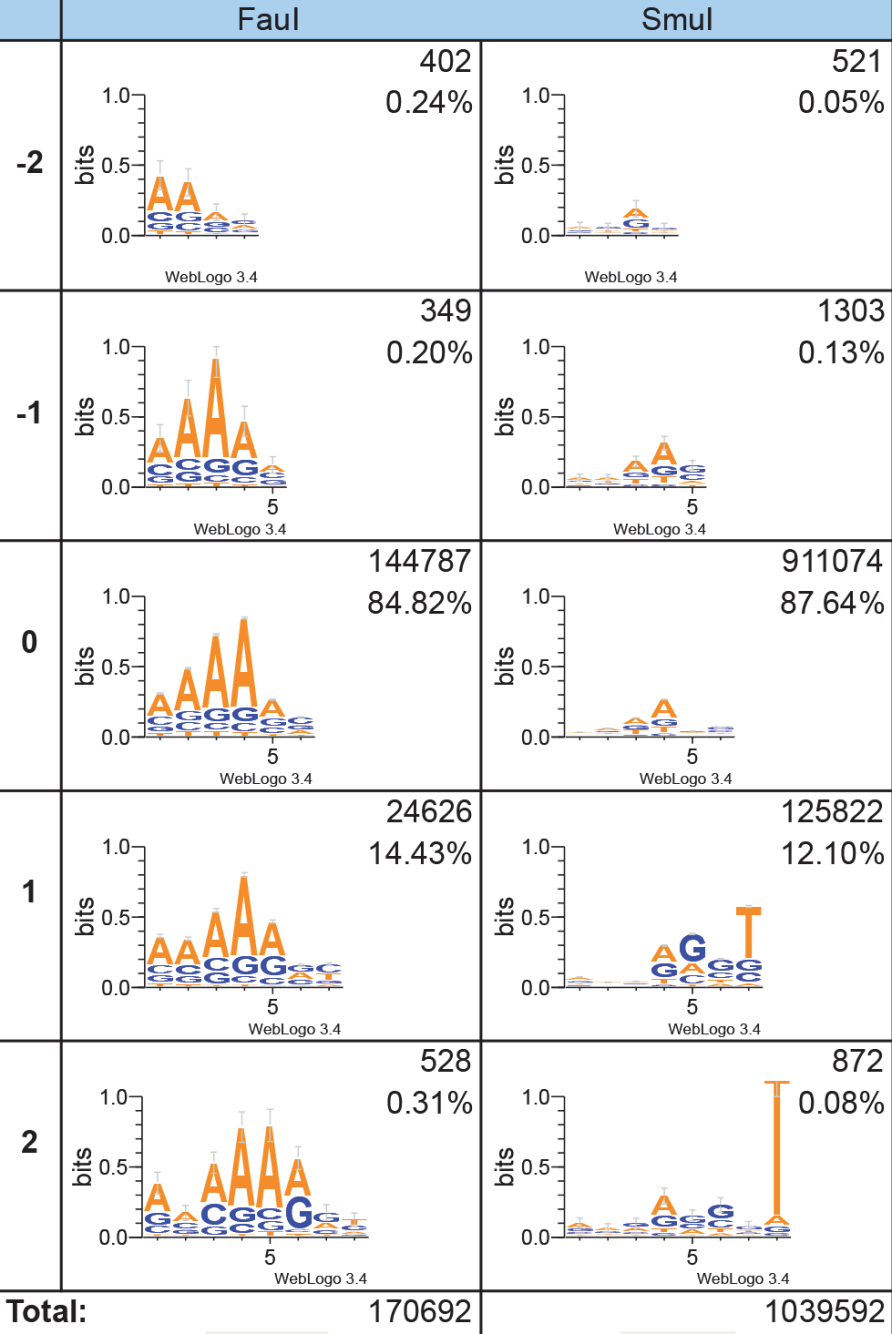
Slippage and sequence dependence of isoschizomers FauI and SmuI. Sequence logos were generated showing the most frequent base per position (in bits) in the region between recognition and cleavage. The detected slippage from −2 to 2 bp are shown independently (row 1 through 5). Recognition site is not included in the plot but was identified immediately left to the plots shown. In the corner the number of sequences identified (and used for the plot) and the relative frequency for that enzyme are shown.

The experimental design also enabled us to investigate the effect on sequence context between the recognition and cleavage sites. This sequence dependency showed a common preference for adenines between the site of recognition and the site for cleavage. A general preference for cytosines proximal to the adapter ligation site is also observed, but not universal (S2–S9 Figs.). As the case for slippage, variation of sequence context is not limited to different REs but also between isoschizomers. This difference is perhaps most striking between FauI and SmuI, where FauI displays a clear preference for adenine-containing substrates while SmuI does not (Fig. 3). The most common slippage of SmuI (+1 bp in 12% of the sequences) also seems to be promoted by a thymine right before the cleavage position where this is not the case for FauI (Fig. 3). The T is even more conserved in the few reads (0.08%) detected with +2 slippage (Fig. 3). For isoschizomers AcuI and Eco57I the sequence dependence is similar, but a slight preference for adenines can be seen for Eco57I and is not present for AcuI (S2 Fig.). Isoschizomers BpmI and GsuI both show similar behaviour. BpmI shows a faint preference for A, whereas GsuI shows no sequence dependence at the preferred length of cleavage. There is a slight bias for cytosine at the site of ligation shared for both of the enzymes (S3 Fig.). BpmI seems to have a stronger dependence on base composition for +1 slippage, with an adenine potentially influencing this behaviour 4 bp away from cleavage (S3 Fig.). This could potentially explain the generally lower frequency of slipped products for BpmI than GsuI. The same enzyme, Eco57MI, showed differing sequence context depending on which recognition sequence was used, where CTGAAG was less dependent on sequence content, and where CTGGAG was slightly inclined to interact with A rich constructs (S4 Fig.). The sequence dependence assay revealed that the +2 slippage of Ecop15I, actually was a −1 bp slippage of a randomly occurring repetition of the recognition sequence (CTGCTGCTG instead of CTGCTG, see S5 Fig.).

### Slippage influencing factors

In this experiment enzyme-to-substrate concentration ratio was kept constant (2U of enzyme per pmol of substrate, except for BsgI, where 1U were used per pmol substrate). Enzyme-to-substrate ratios could potentially influence cleavage effectiveness, e.g. a FokI monomer has been shown to be inactive until dimerising with a second monomer [22, 23]. The varying enzyme concentrations from the suppliers lead to variations in glycerol contents in the reactions used for this experiment (S2 Table). Glycerol concentration has long been known to promote star activity [24, 25], however, no correlation was found between glycerol concentration in the reaction and slippage activity (S11 Fig.) or clear trend for sequence dependence (S2–S9 Figs.). Yet, to further investigate the glycerol factor a subset of five enzymes were selected for a new experiment were the glycerol levels during restriction were kept at 1% (S2 Method, S3–S4 Tables). We can report that a lower glycerol level has a minor non-systematic effect on the slippage levels (S5 Table). We observe both a slippage increase and a slippage decrease and the categorization into low, intermediate and high mode did not change except for BpmI (from low to intermediate) (S5 Table). To validate that the result was not an effect of biased starting material, a follow-up study was conducted where the digestion substrates for the enzymes used to generate the additional dataset were sequenced. Analysis of the randomized region spanning from the recognition to two bases downstream of the digestion site revealed no sequence biases in the starting material (S2 Method, S10 Fig.).

By looking at the 15 enzymes as a group we searched for simple correlations in the dataset, i.e. reaction temperature (S12 Fig.), preferred S-adenosylmethionine (SAM) concentration (S13 Fig.), cleavage distance (S14 Fig.), number of sequence reads obtained (S15 Fig.), and organism source (Table 2), but could not establish any patterns.

### Quality filtering

When all reads were trimmed to 45 bp and any that contained a base with quality score below Q20 (1/100 probability of wrong call) were filtered out, nearly 2.1 million reads remained, and nearly 1.9 million (90.5%) of these were classified as an expected construct and used for slippage analysis. On average the stringent quality filtering resulted in a 0.99 pp shift in slippage. E.g. going from 1.10% to 0.82% total slippage for BseRI (0.28 pp reduction), and from 53.6% to 52.2% for MmeI (1.4 pp reduction) (S6 Table). This indicates a slight inflation of reported slippage due to sequencing errors. Interestingly AcuI exhibited a higher slippage in the quality filtered data (3.35% vs. 1.10% slippage). 3.35% slippage is however more in line with the result gained in the initial approach of analysing the entire read in a search for 10 bp recognition sequence right before the randomized region (total slippage 3.86%) (S6 Table).

## DISCUSSION

The focus of this study was slippage where the general overhang property of the cleavage site is maintained. It is presently unclear if and to what extent the overhangs are variable, termed ‘skewed slippage’. E.g. an overhang of 2 bases could become 3 bases, a 1 base 5’ recessed end could become a 1 base 3’ recessed end, or a blunt end. These variations would not be distinguished with our model system (S16 Fig.) and will need further studies with a slightly revised ligation protocol to characterize. The skewed slippage products are interesting in terms of yield, but would not adversely affect most protocols, as they would not form constructive ends for subsequent ligation. Also, sequence bias upstream of the recognition sequence was not considered in this model system, which might influence the activity of some of REs. Degenerated bases could be added upstream of the recognition site to assay this. For the majority of the RE tested, slippage was determined at constant conditions but, as shown for a subset of the enzymes, the model system could also be used to investigate how certain additives influence the phenomenon. The system is generic and automatable (the extension step can be included by changing the column purification to bead based purification) and many types of potentially influencing factors such as temperature, buffer composition, reagent concentrations, template methylations, etc. warrants future studies. The massively parallel sequencing readout makes the method scalable and sensitive to rare events. Since the effects of all potentially influencing variables was not specifically addressed in this study, it is possible that some of the smaller differences seen between e.g. isoschizomers could be for instance buffer related, rather than related to the enzymes themselves. The wide range of behaviour identified for different enzymes indicates that the phenomenon to a large extent is enzymatic.

The effects observed for Eco57MI, when switching between A or G in the recognition sequence, might also have been influenced by the 2 bp used for isoschizomer differentiation immediately upstream of the recognition sequence (AGCTGAAG and GTCTGGAG). In either case it is interesting that these three alterations seem to influence both slippage and sequence dependence. Further, the original enzyme, Eco57I, showed less slippage than Eco57MI (7% vs. 11% and 13%). Given this, it would be helpful to screen not only for recognition specificity when modifying Type IIS REs but also sequence dependence and slippage. BpuEI has to our knowledge not previously been reported to slip and in this study 40% of products formed were missing one base. We believe this points to a need for general classifications of enzymatic slippage, such as the fidelity index for star activity [18]. Currently we do not have full understanding as to why FauI exhibited such a strong adenine preference. Further studies will be needed to validate and uncover adenine’s influence on efficient digestion with FauI.

Our main hypothesis was that enzymes with longer cleavage distance should demonstrate greater slippage. However, the variation within the set described here varies without a clear link to distance. Enzymes with cleavage distance of 16 bp are found in the low, medium and high slippage range, and the enzymes with shortest distance (FauI and SmuI) both showed intermediate slippage. A possibly inflated average due to the focus on REs with long cleavage distance cannot be excluded, but cleavage distance alone is inadequate as an estimate of slippage, and slippage in general is a highly variable phenomenon. The general lack of simple correlations to slippage in this data between different enzymes makes slippage properties presently hard to predict.

### Previous studies

The enzymes with previous slippage data available (AcuI, MmeI and EcoP15I) agreed well with our findings (Fig. 2)(compare S4 Fig. in Drmanac et al [8] for AcuI and EcoP15I; and S2 Fig. in Shendure et. al [6] for MmeI). The sequence dependence for the endonuclease activity has previously been reported for MmeI [6] and is to our knowledge the only enzyme for which this has been published using an extensive dataset. MmeI showed very little sequence dependence in general in our study, which is concordant with the previous report. The previous study reported on an adenine preference close to the recognition sequence, and reasoned that this could have been introduced during A-tailing when constructing the library. We do not find any preference proximal to the recognition sequence, which supports that conclusion. The previous study was based on mapping reads to a reference genome and found a slight bias downstream of the cleavage site. In our system this potential preference is cleaved away, but the slight bias for cytosine immediately upstream the site of cleavage is supported in our findings, albeit to a higher degree. It is reasonable that a C or G will result in increased ligation efficiency, and is also in general (but not always) the preferred base in the immediate proximity to cleavage in our data.

### Accuracy

The Illumina system is well suited for the task of assaying this type of libraries as (A) it is cost-effective and high throughput, and (B) it has a low insertion-deletion (indel) error frequency. Both of these features contribute to making a sensitive protocol for slippage analysis. The indel rate has been determined to 4 ppm for mapping 100 bp reads to the phiX genome [26]. The impact of sequence quality was investigated by reanalysing the data with a stringent raw data-filtering step. Except for AcuI, this step resulted in a reduction of slippage as compared to the non-filtered analysis, indicating a slightly inflated result, but in general low contribution from sequencing errors.

## Conclusion

The first systematic in-depth study of REs with shifted cleavage sites was performed, and the variation of cleavage shifts (termed slippage) and the sequence dependence thereof were characterised in order to aid future protocol development and understanding of these enzymes. A straightforward, general and automatable model system for studying the activity of REs by using massively parallel sequencing are described, which should be highly applicable for the future studies of large sets of REs and their activity. Slippage is a largely overlooked property of these valuable tools for molecular biology warranting continued study.

## SUPPORTING INFORMATION

S1 Method Oligonucleotides and digestion products. (DOCX)

S2 Method Oligonucleotides and detailed description of generation and analysis of additional dataset. (DOCX)

S1 Fig Bar plots showing number of reads per cleavage position. Generally low background from unspecific/random detection is observed.
(TIF)

S2 Fig Sequence logos for all sequences detected for isoschizomers AcuI and Eco57I within +/− 2 bp of the expected cleavage distance. In grey is (in order from top) the type of overhang produced, number of sequences detected for that length and percent of total sequences detected within +/− 2 bp. Few sequences were detected at 2 bp distance, which make the sequence logos uncertain for those lengths.
(TIF)

S3 Fig Sequence logos for all sequences detected for isoschizomers BpmI and GsuI within +/− 2 bp of the expected cleavage distance. In grey is (in order from top) the type of overhang produced, number of sequences detected for that length and percent of total sequences detected within +/− 2 bp. Few sequences were detected at 2 bp distance, which make the sequence logos uncertain for those lengths.
(TIF)

S4 Fig Sequence logos for all sequences detected for Eco57MI within +/− 2 bp of the expected cleavage distance. Two substrates were used to assay differences in A and G bases in the recognition sequence. In grey is (in order from top) the type of overhang produced, number of sequences detected for that length and percent of total sequences detected within +/− 2 bp. Few sequences were detected at 2 bp distance, which make the sequence logos uncertain for those lengths.
(TIF)

S5 Fig Sequence logos for all sequences detected for EcoP15I within +/− 2 bp of the expected cleavage distance. In grey is (in order from top) the type of overhang produced, number of sequences detected for that length and percent of total sequences detected within +/− 2 bp. Few sequences were detected at 2 bp distance, which make the sequence logos uncertain for those lengths. For this enzyme, a conserved motif “CAG” immediately proximal to the recognition sequence (CAGCAG) means the +2 detected here is actually a −1 slippage of a recognition sequence shifted three bases downstream.
(TIF)

S6 Fig Sequence logos for all sequences detected for BpuEI within +/− 2 bp of the expected cleavage distance. In grey is (in order from top) the type of overhang produced, number of sequences detected for that length and percent of total sequences detected within +/− 2 bp. Few sequences were detected at 2 bp distance, which make the sequence logos uncertain for those lengths.
(TIF)

S7 Fig Sequence logos for all sequences detected for FokI within +/− 2 bp of the expected cleavage distance. In grey is (in order from top) the type of overhang produced, number of sequences detected for that length and percent of total sequences detected within +/− 2 bp. Few sequences were detected at 2 bp distance, which make the sequence logos uncertain for those lengths.
(TIF)

S8 Fig Sequence logos for all sequences detected for MmeI within +/− 2 bp of the expected cleavage distance. In grey is (in order from top) the type of overhang produced, number of sequences detected for that length and percent of total sequences detected within +/− 2 bp. Few sequences were detected at 2 bp distance, which make the sequence logos uncertain for those lengths.
(TIF)

S9 Fig Sequence logos for all sequences detected for BbvI within +/− 2 bp of the expected cleavage distance. In grey is (in order from top) the type of overhang produced, number of sequences detected for that length and percent of total sequences detected within +/− 2 bp. Few sequences were detected at 2 bp distance, which make the sequence logos uncertain for those lengths.
(TIF)

S10 Fig Sequence logos for the substrates used to generate the additional dataset. (TIF)

S11 Fig Glycerol concentration plotted over total slippage detected for each enzyme. (PNG)

S12 Fig Reaction temperature plotted over total slippage detected for each enzyme. (PNG)

S13 Fig S-adenosylmethionine (SAM) concentration used for reactions plotted over total slippage detected for each enzyme. (PNG)

S14 Fig Cleavage distance plotted over total slippage detected. (PNG)

S15 Fig Number of reads obtained for each enzyme plotted over final glycerol concentration in that sample. (PNG)

S16 Fig Skewed-slippage. Although our model system was not designed to detect skewed slippage, two kinds of skewed product will give product. Since only the bottom strand is used for detection, bottom slippage of +1 and top slippage of −1 can form productive constructs, as indicated in the red box. Plus 1 bottom slippage is detected as +1 slippage, and −1 top slippage is detected as no slippage (false negative). These products would be disfavoured during ligation due to the gapped position and we expect these events, if they exist, to be low.
(PNG)

S1 Table Total slippage detected for all enzymes assayed. (DOCX)

S2 Table Enzyme storage and reaction buffer additions for the primary dataset. The effects of storage conditions for each enzyme is shown added to 1x of the recommended reaction buffers. The final reaction conditions (not including supplements) depending on the amount of enzyme used for each reaction is shown.
(DOCX)

S3 Table Enzyme storage and reaction buffer additions for the additional dataset. The effects of storage conditions for each enzyme is shown added to 1x of the recommended reaction buffers. The final reaction conditions (not including supplements) depending on the amount of enzyme used for each reaction is shown.
(DOCX)

S4 Table The different reaction conditions for each enzyme in the additional dataset. (DOCX)

S5 Table Total slippage detected for all enzymes in the additional dataset. The additional dataset was analysed for slippage events within +/− 2 bp from the expected distance. To assess the impact of sequence errors a quality dataset (ca. 1.1 million 45 bp reads where all bases had quality scores above 20. i.e. less than 1% chance of sequencing error) was selected.
(DOCX)

S6 Table The total amount of slippage detected +/− 2 bp away from the recognition sequence. The dataset was analysed in three different ways to assay robustness. The “expected distance” was used as the primary analysis as it was judged to be most sensitive. “Entire read” analysis is based on perfect matches of a 10 bp recognition sequence right before the randomized position. No consideration was taken to at what distance the 10 bp matched. “Quality filtered” used on a limited high quality dataset (ca. 2 million 45 bp reads where all bases had quality scores above 20, i.e. less than 1% chance of sequencing error). Expected distance assumed the recognition sequence to be +/− 2 bp away from the recognition sequence and searched in order from longest to shortest distance for each read with a perfect match to a unique sequence for each enzyme. The results are similar and does not alter the general pattern identified, but can be viewed as an indication of the level of the sequencing assay induced uncertainty.
(DOCX)
