# Supplementary material for: Endonuclease Specificity and Sequence Dependence of Type IIS Restriction Enzymes
Source: PLoS One. 2015 Jan 28;10(1):e0117059. doi: 10.1371/journal.pone.0117059 (PMC4309577; doi:10.1371/journal.pone.0117059)
Supplement: S1 Method — (DOCX) [file pone.0117059.s001.docx]

**Supporting methods S1**

**Model system design**

The different sequence elements are numbered using Roman numerals and represents the following:

1. Illumina primer (34 nts)
2. Spacer I. The purpose of this is to differentiate the length of the digestion product from the adapter length. The spacer contains an EcoRI site (15 nts)
3. GsuI recognition site. Two additional nucleotides to differentiate isoschizomers. (8 nts)
4. Random sequence (18 nts)
5. Spacer containing a StuI site (6 nts)
6. Spacer II. In order to create a distance between the digestion site and the end of the fragment (12 nts)
7. 3’ NN overhang (2 nts)
8. Treatment tag (2-nt 3’ OH) (6 nts)
9. Illumina hyb sequence and sequencing primer (58)

GsuI substrate (and infill primer)

5’- CAAGCAGAAGACGGCATACG -3’

3’- GTTCGTCTTCTGCCGTATGCTCGAGAAGGCTAGA CTTAAGACATTCGCC CGGACCTC NNNNNNNNNNNNNNNNNN TCCGGA AGTGCTAGGTCT -5’

I II III IV V VI

BpmI substrate

3’- GTTCGTCTTCTGCCGTATGCTCGAGAAGGCTAGA CTTAAGACATTCGCC TAGACCTC NNNNNNNNNNNNNNNNNN TCCGGA AGTGCTAGGTCT -5’

BsgI substrate

3’- GTTCGTCTTCTGCCGTATGCTCGAGAAGGCTAGA CTTAAGACATTCGCC TACACGTC NNNNNNNNNNNNNNNNNN TCCGGA AGTGCTAGGTCT -5’

BpuEI substrate

3’- GTTCGTCTTCTGCCGTATGCTCGAGAAGGCTAGA CTTAAGACATTCGCC TAGAACTC NNNNNNNNNNNNNNNNNN TCCGGA AGTGCTAGGTCT -5’

Eco57I substrate

3’- GTTCGTCTTCTGCCGTATGCTCGAGAAGGCTAGA CTTAAGACATTCGCC CCGACTTC NNNNNNNNNNNNNNNNNN TCCGGA AGTGCTAGGTCT -5’

Eco57MI-a substrate

3’- GTTCGTCTTCTGCCGTATGCTCGAGAAGGCTAGA CTTAAGACATTCGCC TCGACTTC NNNNNNNNNNNNNNNNNN TCCGGA AGTGCTAGGTCT -5’

Eco57MI-g substrate

3’- GTTCGTCTTCTGCCGTATGCTCGAGAAGGCTAGA CTTAAGACATTCGCC CAGACCTC NNNNNNNNNNNNNNNNNN TCCGGA AGTGCTAGGTCT -5’

AcuI substrate

3’- GTTCGTCTTCTGCCGTATGCTCGAGAAGGCTAGA CTTAAGACATTCGCC TTGACTTC NNNNNNNNNNNNNNNNNN TCCGGA AGTGCTAGGTCT -5’

MmeI substrate

3’- GTTCGTCTTCTGCCGTATGCTCGAGAAGGCTAGA CTTAAGACATTCGCC TTAGGTTG NNNNNNNNNNNNNNNNNNNNNN TCCGGA AGTGCTAGGTCT -5’

BseRI substrate

3’- GTTCGTCTTCTGCCGTATGCTCGAGAAGGCTAGA CTTAAGACATTCGCC TTCTCCTC NNNNNNNNNNNN TCCGGA AGTGCTAGGTCT -5’

BbvI substrate

3’- GTTCGTCTTCTGCCGTATGCTCGAGAAGGCTAGA CTTAAGACATTCGCC TTCCGTCG NNNNNNNNNNNNNN TCCGGA AGTGCTAGGTCT -5’

FokI substrate

3’- GTTCGTCTTCTGCCGTATGCTCGAGAAGGCTAGA CTTAAGACATTCGCC TTCCCTAC NNNNNNNNNNNNNNN TCCGGA AGTGCTAGGTCT -5’

FauI substrate

3’- GTTCGTCTTCTGCCGTATGCTCGAGAAGGCTAGA CTTAAGACATTCGCC TCAGGGCG NNNNNNNN TCCGGA AGTGCTAGGTCT -5’

SmuI substrate

3’- GTTCGTCTTCTGCCGTATGCTCGAGAAGGCTAGA CTTAAGACATTCGCC TTCGGGCG NNNNNNNN TCCGGA AGTGCTAGGTCT -5’

EcoP15I substrate

3’- GTTCGTCTTCTGCCGTATGCTCGAGAAGGCTAGA CTTAAG GACGACGTCGTC NNNNNNNNNNNNNNNNNNNNNNNNNNNNN TCCGGA AGTGCTAGGTCT -5’

**Adapters**

Phosphate

A1-3’-NN adapter (5’-PHO)

5’- AATGATACGGCGACCACCGAGATCTACACTCTTTCCCTACACGACGCTCTTCCGATCT GCTGCT NN -3’ (66/64)

3’- TTACTATGCCGCTGGTGGCTCTAGATGTGAGAAAGGGATGTGCTGCGAGAAGGCTAGA CGACGA -5’

IX VIII VII

A2-5’-NN adapter (5’-PHO)

5’- AATGATACGGCGACCACCGAGATCTACACTCTTTCCCTACACGACGCTCTTCCGATCT AAGGTA -3’ (64/66)

3’- TTACTATGCCGCTGGTGGCTCTAGATGTGAGAAAGGGATGTGCTGCGAGAAGGCTAGA TTCCAT NN -5’

A3-5’-NNNN adapter (5’-PHO)

5’- AATGATACGGCGACCACCGAGATCTACACTCTTTCCCTACACGACGCTCTTCCGATCT AGCGCT -3’ (64/68)

3’- TTACTATGCCGCTGGTGGCTCTAGATGTGAGAAAGGGATGTGCTGCGAGAAGGCTAGA TCGCGA NNNN -5’

A4-5’-NN adapter for EcoP15I (5’-PHO)

5’- AATGATACGGCGACCACCGAGATCTACACTCTTTCCCTACACGACGCTCTTCCGATCT CTTTAG -3’ (64/66)

3’- TTACTATGCCGCTGGTGGCTCTAGATGTGAGAAAGGGATGTGCTGCGAGAAGGCTAGA GAAATC NN -5’

**Oligos for the primary dataset**

**Primers**

P1, Infill Primer, 5’-Biotin

5’- CAAGCAGAAGACGGCATACG -3’

P2-1832, Fwd primer

5’- CAAGCAGAAGACGGCATACG -3’

P3-1833, Rev primer

5’- AATGATACGGCGACCACCG – 3’

**Template oligonucleotides**

T1 GsuI substratE

5’- TCTGGATCGTGAAGGCCTNNNNNNNNNNNNNNNNNNCTCCAGGCCCGCTTACAGAATTCAGATCGGAAGAGCTCGTATGCCGTCTTCTGCTTG -3’

T2 BpmI substrate

5’- TCTGGATCGTGAAGGCCTNNNNNNNNNNNNNNNNNNCTCCAGATCCGCTTACAGAATTCAGATCGGAAGAGCTCGTATGCCGTCTTCTGCTTG -3’

T3 BsgI substrate

5’- TCTGGATCGTGAAGGCCTNNNNNNNNNNNNNNNNNNCTGCACATCCGCTTACAGAATTCAGATCGGAAGAGCTCGTATGCCGTCTTCTGCTTG -3’

T4 BpuEI substrate

5’- TCTGGATCGTGAAGGCCTNNNNNNNNNNNNNNNNNNCTCAAGATCCGCTTACAGAATTCAGATCGGAAGAGCTCGTATGCCGTCTTCTGCTTG -3’

T5 Eco57I substrate

5’- TCTGGATCGTGAAGGCCTNNNNNNNNNNNNNNNNNNCTTCAGCCCCGCTTACAGAATTCAGATCGGAAGAGCTCGTATGCCGTCTTCTGCTTG -3’

T6 Eco57MI-a substrate

5’- TCTGGATCGTGAAGGCCTNNNNNNNNNNNNNNNNNNCTTCAGCTCCGCTTACAGAATTCAGATCGGAAGAGCTCGTATGCCGTCTTCTGCTTG -3’

T7 Eco57MI-g substrate

5’- TCTGGATCGTGAAGGCCTNNNNNNNNNNNNNNNNNNCTCCAGACCCGCTTACAGAATTCAGATCGGAAGAGCTCGTATGCCGTCTTCTGCTTG -3’

T8 AcuI substrate

5’- TCTGGATCGTGAAGGCCTNNNNNNNNNNNNNNNNNNCTTCAGTTCCGCTTACAGAATTCAGATCGGAAGAGCTCGTATGCCGTCTTCTGCTTG -3’

T9 MmeI substrate

5’- TCTGGATCGTGAAGGCCTNNNNNNNNNNNNNNNNNNNNNNGTTGGATTCCGCTTACAGAATTCAGATCGGAAGAGCTCGTATGCCGTCTTCTGCTTG -3’

T10 BseRI substrate

5’- TCTGGATCGTGAAGGCCTNNNNNNNNNNNNCTCCTCTTCCGCTTACAGAATTCAGATCGGAAGAGCTCGTATGCCGTCTTCTGCTTG -3’

T11 BbvI substrate

5’- TCTGGATCGTGAAGGCCTNNNNNNNNNNNNNNGCTGCCTTCCGCTTACAGAATTCAGATCGGAAGAGCTCGTATGCCGTCTTCTGCTTG -3’

T12 FokI substrate

5’- TCTGGATCGTGAAGGCCTNNNNNNNNNNNNNNNCATCCCTTCCGCTTACAGAATTCAGATCGGAAGAGCTCGTATGCCGTCTTCTGCTTG -3’

T13 FauI substrate

5’- TCTGGATCGTGAAGGCCTNNNNNNNNGCGGGACTCCGCTTACAGAATTCAGATCGGAAGAGCTCGTATGCCGTCTTCTGCTTG -3’

T14 SmuI substrate

5’- TCTGGATCGTGAAGGCCTNNNNNNNNGCGGGCTTCCGCTTACAGAATTCAGATCGGAAGAGCTCGTATGCCGTCTTCTGCTTG-3’

T15 EcoP15I (t2t) substrate

5’- TCTGGATCGTGAAGGCCTNNNNNNNNNNNNNNNNNNNNNNNNNNNNN<CTGCTG|CAGCAG>GAATTCAGATCGGAAGAGCTCGTATGCCGTCTTCTGCTTG-3’

**Adapters**

NN adapter sense

5’- AATGATACGGCGACCACCGAGATCTACACTCTTTCCCTACACGACGCTCTTCCGATCTGCTGCTNN -3’

NN adapter antisense, 5’-phosphate

5’- AGCAGCAGATCGGAAGAGCGTCGTGTAGGGAAAGAGTGTAGATCTCGGTGGTCGCCGTATCATT -3’

5’-NN adapter sense

5’- AATGATACGGCGACCACCGAGATCTACACTCTTTCCCTACACGACGCTCTTCCGATCTAAGGTA -3’

5’-NN adapter antisense (5’-PHO)

5’- NNTACCTTAGATCGGAAGAGCGTCGTGTAGGGAAAGAGTGTAGATCTCGGTGGTCGCCGTATCATT -3’

5’-NNNN adapter sense

5’- AATGATACGGCGACCACCGAGATCTACACTCTTTCCCTACACGACGCTCTTCCGATCTAGCGCT -3’

5’-NNNN adapter antisense (5’-PHO)

5’- NNNNAGCGCTAGATCGGAAGAGCGTCGTGTAGGGAAAGAGTGTAGATCTCGGTGGTCGCCGTATCATT -3’

5’-NN adapter for EcoP15I sense

5’- AATGATACGGCGACCACCGAGATCTACACTCTTTCCCTACACGACGCTCTTCCGATCTCTTTAG -3’

5’-NN adapter for EcoP15I antisense (5’-PHO)

5’- NNCTAAAGAGATCGGAAGAGCGTCGTGTAGGGAAAGAGTGTAGATCTCGGTGGTCGCCGTATCATT -3’

**Digestion products**

Biotin, Recognition site

**GsuI substrate** (93bp)

5’- TCTGGATCGTGAAGGCCTNNNN NNNNNNNNNNNNNNCTCCAGGCCCGCTTACAGAATTCAGATCGGAAGAGCTCGTATGCCGTCTTCTGCTTG -3’

3’- AGACCTAGCACTTCCGGANN NNNNNNNNNNNNNNNNGAGGTCCGGGCGAATGTCTTAAGTCTAGCCTTCTCGAGCATACGGCAGAAGACGAAC -5’

(22/20) (71/73)

**BpmI substrate** (93bp)

5’- TCTGGATCGTGAAGGCCTNNNN NNNNNNNNNNNNNNCTCCAGATCCGCTTACAGAATTCAGATCGGAAGAGCTCGTATGCCGTCTTCTGCTTG -3’

3’- AGACCTAGCACTTCCGGANN NNNNNNNNNNNNNNNNGAGGTCTAGGCGAATGTCTTAAGTCTAGCCTTCTCGAGCATACGGCAGAAGACGAAC -5’

(22/20) (71/73)

**BsgI substrate** (93bp)

5’- TCTGGATCGTGAAGGCCTNNNN NNNNNNNNNNNNNNCTGCACATCCGCTTACAGAATTCAGATCGGAAGAGCTCGTATGCCGTCTTCTGCTTG -3’

3’- AGACCTAGCACTTCCGGANN NNNNNNNNNNNNNNNNGACGTGTAGGCGAATGTCTTAAGTCTAGCCTTCTCGAGCATACGGCAGAAGACGAAC -5’

(22/20) (71/73)

**BpuEI substrate** (93bp)

5’- TCTGGATCGTGAAGGCCTNNNN NNNNNNNNNNNNNNCTCAAGATCCGCTTACAGAATTCAGATCGGAAGAGCTCGTATGCCGTCTTCTGCTTG -3’

3’- AGACCTAGCACTTCCGGANN NNNNNNNNNNNNNNNNGAGTTCTAGGCGAATGTCTTAAGTCTAGCCTTCTCGAGCATACGGCAGAAGACGAAC -5’

(22/20) (71/73)

**Eco57I substrate** (93bp)

5’- TCTGGATCGTGAAGGCCTNNNN NNNNNNNNNNNNNNCTTCAGCCCCGCTTACAGAATTCAGATCGGAAGAGCTCGTATGCCGTCTTCTGCTTG -3’

3’- AGACCTAGCACTTCCGGANN NNNNNNNNNNNNNNNNGAAGTCGGGGCGAATGTCTTAAGTCTAGCCTTCTCGAGCATACGGCAGAAGACGAAC -5’

(22/20) (71/73)

**Eco57MI-a substrate** (93bp)

5’- TCTGGATCGTGAAGGCCTNNNN NNNNNNNNNNNNNNCTTCAGCTCCGCTTACAGAATTCAGATCGGAAGAGCTCGTATGCCGTCTTCTGCTTG -3’

3’- AGACCTAGCACTTCCGGANN NNNNNNNNNNNNNNNNGAAGTCGAGGCGAATGTCTTAAGTCTAGCCTTCTCGAGCATACGGCAGAAGACGAAC -5’

(22/20) (71/73)

**Eco57MI-g substrate** (93bp)

5’- TCTGGATCGTGAAGGCCTNNNN NNNNNNNNNNNNNNCTCCAGACCCGCTTACAGAATTCAGATCGGAAGAGCTCGTATGCCGTCTTCTGCTTG -3’

3’- AGACCTAGCACTTCCGGANN NNNNNNNNNNNNNNNNGAGGTCTGGGCGAATGTCTTAAGTCTAGCCTTCTCGAGCATACGGCAGAAGACGAAC -5’

(22/20) (71/73)

**AcuI substrate** (93bps)

5’- TCTGGATCGTGAAGGCCTNNNN NNNNNNNNNNNNNNCTTCAGTTCCGCTTACAGAATTCAGATCGGAAGAGCTCGTATGCCGTCTTCTGCTTG -3’

3’- AGACCTAGCACTTCCGGANN NNNNNNNNNNNNNNNNGAAGTCAAGGCGAATGTCTTAAGTCTAGCCTTCTCGAGCATACGGCAGAAGACGAAC -5’

(22/20) (71/73)

**MmeI substrate** (97bps)

5’- TCTGGATCGTGAAGGCCTNNNN NNNNNNNNNNNNNNNNNNGTTGGATTCCGCTTACAGAATTCAGATCGGAAGAGCTCGTATGCCGTCTTCTGCTTG -3’

3’- AGACCTAGCACTTCCGGANN NNNNNNNNNNNNNNNNNNNNCAACCTAAGGCGAATGTCTTAAGTCTAGCCTTCTCGAGCATACGGCAGAAGACGAAC -5’

(22/20) (75/77)

**BseRI substrate** (87bps)

5’- TCTGGATCGTGAAGGCCTNNNN NNNNNNNNCTCCTCTTCCGCTTACAGAATTCAGATCGGAAGAGCTCGTATGCCGTCTTCTGCTTG -3’

3’- AGACCTAGCACTTCCGGANN NNNNNNNNNNGAGGAGAAGGCGAATGTCTTAAGTCTAGCCTTCTCGAGCATACGGCAGAAGACGAAC -5’

(22/20) (65/67)

**BbvI substrate** (89bps)

5’- TCTGGATCGTGAAGGCCTNN NNNNNNNNNNNNGCTGCCTTCCGCTTACAGAATTCAGATCGGAAGAGCTCGTATGCCGTCTTCTGCTTG -3’

3’- AGACCTAGCACTTCCGGANNNNNN NNNNNNNNCGACGGAAGGCGAATGTCTTAAGTCTAGCCTTCTCGAGCATACGGCAGAAGACGAAC -5’

(20/24) (69/65)

**FokI substrate** (90bps)

5’- TCTGGATCGTGAAGGCCTNN NNNNNNNNNNNNNCATCCCTTCCGCTTACAGAATTCAGATCGGAAGAGCTCGTATGCCGTCTTCTGCTTG -3’

3’- AGACCTAGCACTTCCGGANNNNNN NNNNNNNNNGTAGGGAAGGCGAATGTCTTAAGTCTAGCCTTCTCGAGCATACGGCAGAAGACGAAC -5’

(20/24) (70/66)

**FauI substrate** (83bps)

5’- TCTGGATCGTGAAGGCCTNN NNNNNNGCGGGACTCCGCTTACAGAATTCAGATCGGAAGAGCTCGTATGCCGTCTTCTGCTTG -3’

3’- AGACCTAGCACTTCCGGANNNN NNNNCGCCCTGAGGCGAATGTCTTAAGTCTAGCCTTCTCGAGCATACGGCAGAAGACGAAC -5’

(20/22) (63/61)

**SmuI substrate** (83bps)

5’- TCTGGATCGTGAAGGCCTNN NNNNNNGCGGGCTTCCGCTTACAGAATTCAGATCGGAAGAGCTCGTATGCCGTCTTCTGCTTG -3’

3’- AGACCTAGCACTTCCGGANNNN NNNNCGCCCGAAGGCGAATGTCTTAAGTCTAGCCTTCTCGAGCATACGGCAGAAGACGAAC -5’

(20/22) (63/31)

**EcoP15I substrate** (99bps)

5’- TCTGGATCGTGAAGGCCTNN NNNNNNNNNNNNNNNNNNNNNNNNNNNCTGCTGCAGCAGGAATTCAGATCGGAAGAGCTCGTATGCCGTCTTCTGCTTG -3’

3’- AGACCTAGCACTTCCGGANNNN NNNNNNNNNNNNNNNNNNNNNNNNNGACGACGTCGTCCTTAAGTCTAGCCTTCTCGAGCATACGGCAGAAGACGAAC -5’

(20/22) (79/77)
