# Supplementary material for: Endonuclease Specificity and Sequence Dependence of Type IIS Restriction Enzymes
Source: PLoS One. 2015 Jan 28;10(1):e0117059. doi: 10.1371/journal.pone.0117059 (PMC4309577; doi:10.1371/journal.pone.0117059)
Supplement: S2 Method — (DOCX) [file pone.0117059.s002.docx]

**Supporting methods S2**

**Oligos for additional dataset**

The oligonucleotides used for the additional dataset was slightly altered to allow for sample indexing. Reverse PCR primer and adapters were identical to the ones used for the primary dataset (Method S1)

**Primers**

SP1, Infill Primer, 5’-Biotin

5’- GTGACTGGAGTTCAGACGTG -3’

SP2, Fwd primer, index 25

5’- CAAGCAGAAGACGGCATACGAGATACTGATGTGACTGGAGTTC -3’

SP3, Re primer with Illumina adaptor sequence

5’- AATGATACGGCGACCACCGAGATCTACACTCTTTCCCTACACGACGCTCTTCCGATCTTCTGGATCGTGTAGGCCT -3’

**Template oligonucleotides**

ST1, GsuI substrate

5’-TCTGGATCGTGAAGGCCTNNNNNNNNNNNNNNNNNNCTCCAGGCCCGCTTACAGAATTCAGATCGGAAGAGCACACGTCTGAACTCCAGTCAC-3’

ST2, BpmI substrate

5’-TCTGGATCGTGAAGGCCTNNNNNNNNNNNNNNNNNNCTCCAGATCCGCTTACAGAATTCAGATCGGAAGAGCACACGTCTGAACTCCAGTCAC-3’

ST3, BpuEI substrate

5’-TCTGGATCGTGAAGGCCTNNNNNNNNNNNNNNNNNNCTCAAGATCCGCTTACAGAATTCAGATCGGAAGAGCACACGTCTGAACTCCAGTCAC-3’

ST4, MmeI substrate

5’-TCTGGATCGTGAAGGCCTNNNNNNNNNNNNNNNNNNNNNNGTTGGATTCCGCTTACAGAATTCAGATCGGAAGAGCACACGTCTGAACTCCAGTCAC-3’

ST5, BbvI substrate

5’-TCTGGATCGTGAAGGCCTNNNNNNNNNNNNNNGCTGCCTTCCGCTTACAGAATTCAGATCGGAAGAGCACACGTCTGAACTCCAGTCAC-3’

**Generation of additional datasets**

In order to further investigate whether the acquired result was influenced by the reaction conditions, a subset of five enzymes (BbvI, BpmI, BpuEI, GsuI and MmeI) were select for a second experiment. The subset was selected to include enzymes from all three classes of slippage, low slippage (0-2%), intermediate slippage (2-15%), and high slippage (>15%). For this assay a fixed enzyme volume (1 µl) was used for digestion of 20 pmol of substrate. The fixed volume resulted in varying amounts of enzyme in the reactions due to the different stock concentrations of the enzymes. This lead to that the enzyme storage buffer had less of an effect on the final reaction buffer composition (compare Table S2 and Table S3). A shortage of restriction enzymes should not skew the slippage tendencies negatively, but simply leading to less digested products.

The sequencing library was prepared using the same protocol as used for the original library, with the following modifications: Altered oligonucleotide sequences to allow for sample indexing (SP1, SP2, ST1-ST5), a lower enzyme-to-substrate ratio (Table S4) and 25 cycles of PCR.

The substrate was also sequenced to further assess potential biases in the starting material. The sequencing library was prepared as follows. Two 50 µl PCR reactions in 1x Phusion High-Fidelity PCR Master Mix containing 2 pmol digestion substrate 500 nM SP2 and SP3 were performed for each substrate. The PCR was run at 98°C 30 seconds, [98°C 10 seconds, 62°C 30 seconds, 72°C 15 seconds] x 13 cycles, 72°C 5 min, 4°C hold. The two reactions of each sample were pooled and concentrated using MinElute Spin columns (Qiagen).

The libraries were sequenced on the Illumina HiSeq 2500 at National Genomics Infrastructure (NGI) (Sweden). Raw sequencing reads are available at the NCBI Sequence Read Archive under accession SRS627913

**Results from additional dataset**

4,051,280 reads were obtained from the additional experiment, of those 3,967,273 (97.9%) reads contained a ligation adapter and for 2,362,142 (58.3%) of those, a recognition sequence corresponding to one of the surveyed enzymes could be found.

The data was analysed in the same way as the primary data using a custom python script. The two restriction enzymes most prone to slippage were BpuEI and MmeI with a total slippage of 34.6% and 49.9% respectively. They thus remained in the high slippage class despite the altered reaction conditions. GsuI showed a slightly higher slippage tendency in the second slippage experiment (from 5% to 7.4%), but still remained in the intermediate slippage class (Table S5). BbvI and BpmI both originated from the low slippage class, but the new slippage level of BpmI increased enough to place it among the intermediate slippage enzymes. BbvI remained in the low slippage class.

The primary assay, with a fixed enzyme to substrate ratio, conveyed a high level of glycerol in several of the reactions. High glycerol risks relaxing the recognition, leading to star activity. It is not clear as to what extent glycerol affects slippage tendencies in type IIS restriction enzymes and a clear correlation was not apparent in this data. The altered assay meant a glycerol level of 1%, but this did not translate into that all of the enzymes performed better under these conditions. Though relative few reads the slippage tendencies were higher for GsuI and BpmI in the altered assay.

Using a custom Python script the reads from the substrate sequencing were counted and mapped to respective enzyme based on their enzyme recognition sequence. The randomer regions were extracted and used to generate sequence logos (Weblogo 3.4) (Figure S10). No sequence bias could be observed for any of the substrates.
