# Supplementary figures and images for: Endonuclease Specificity and Sequence Dependence of Type IIS Restriction Enzymes
Source: PLoS One. 2015 Jan 28;10(1):e0117059. doi: 10.1371/journal.pone.0117059 (PMC4309577; doi:10.1371/journal.pone.0117059)

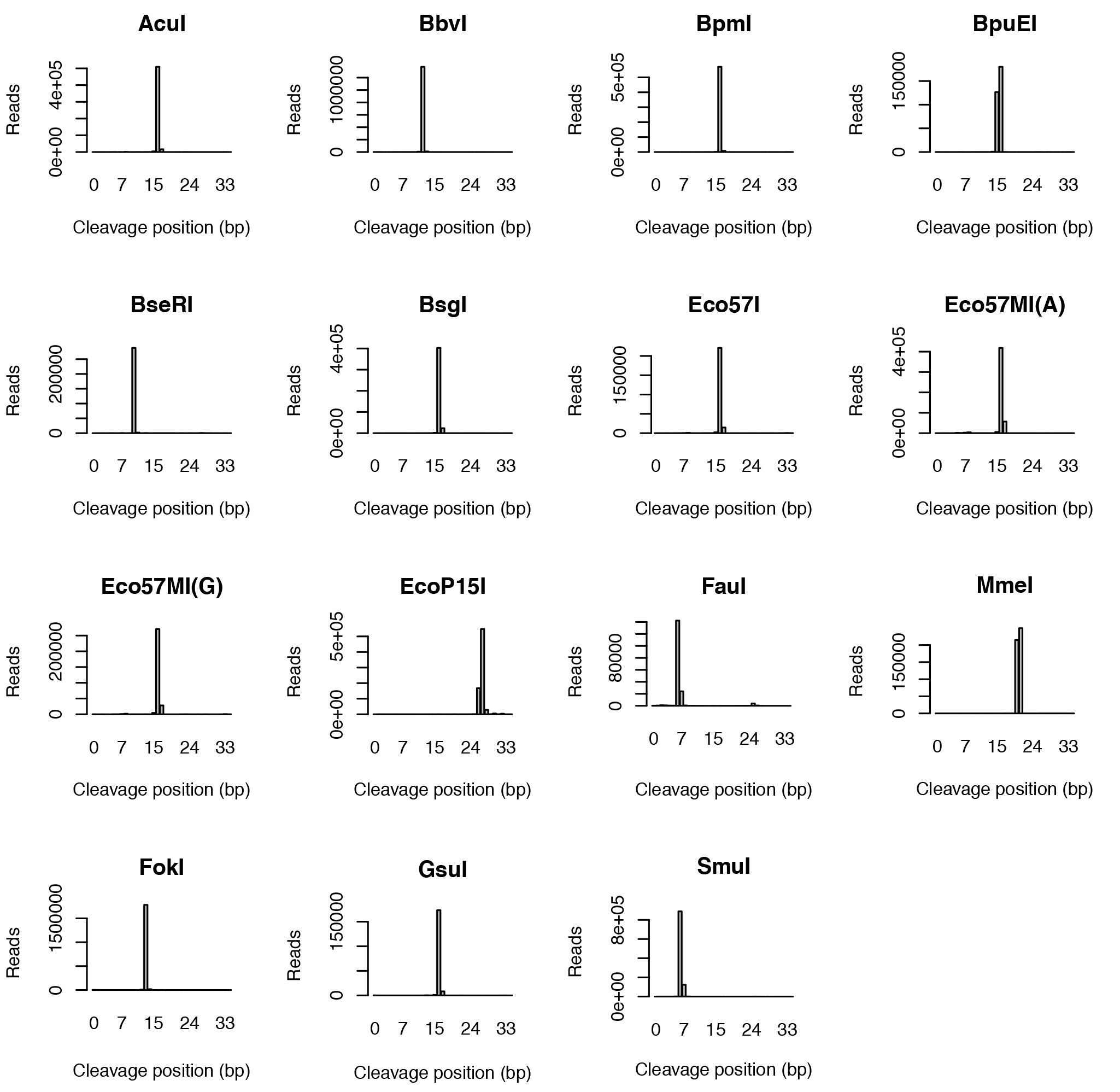

Supplement: S1 Fig — Generally low background from unspecific/random detection is observed. (TIF) [file pone.0117059.s003.tif]

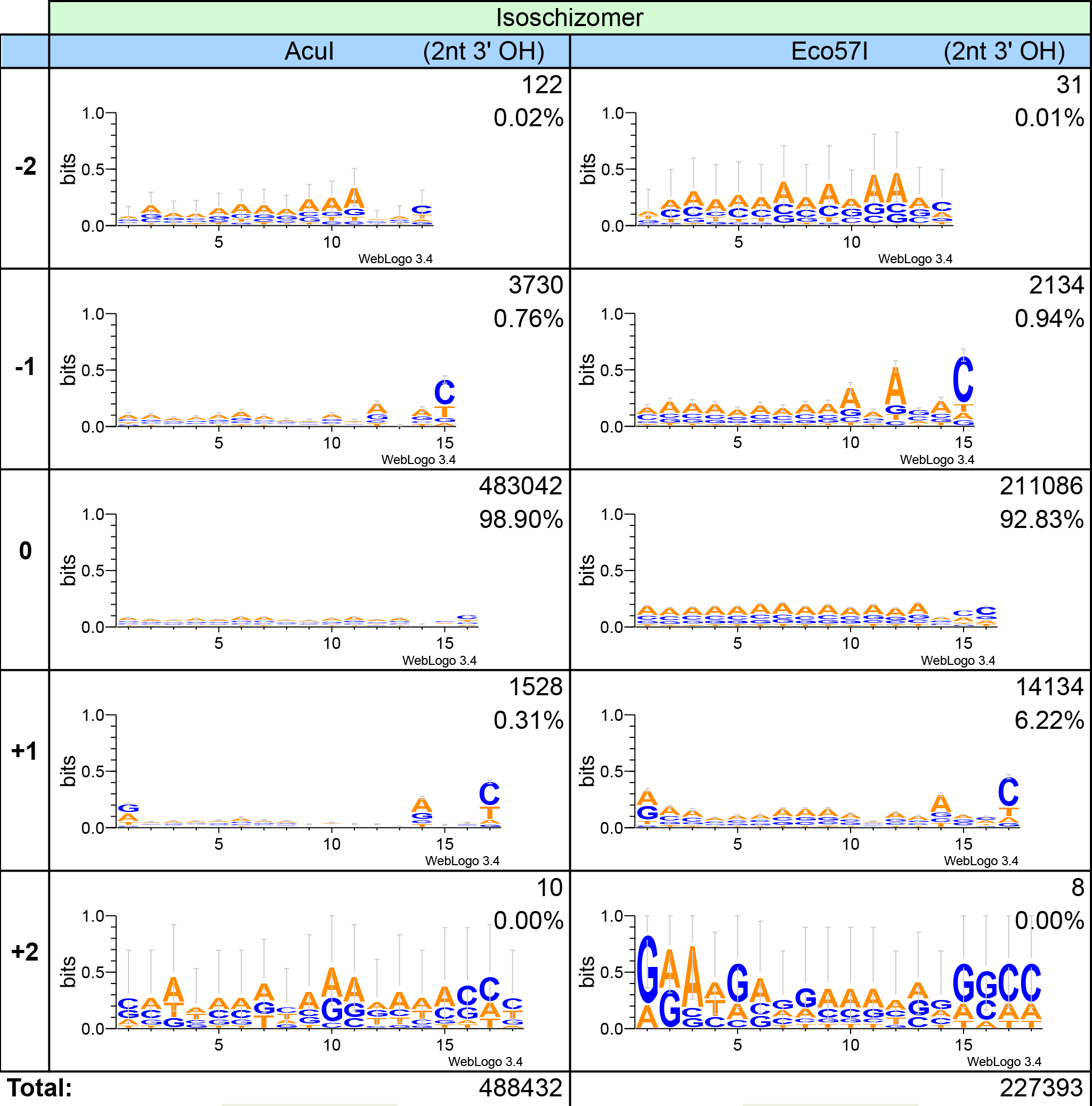

Supplement: S2 Fig — In grey is (in order from top) the type of overhang produced, number of sequences detected for that length and percent of total sequences detected within +/− 2 bp. Few sequences were detected at 2 bp distance, which make the sequence logos uncertain for those lengths. (TIF) [file pone.0117059.s004.tif]

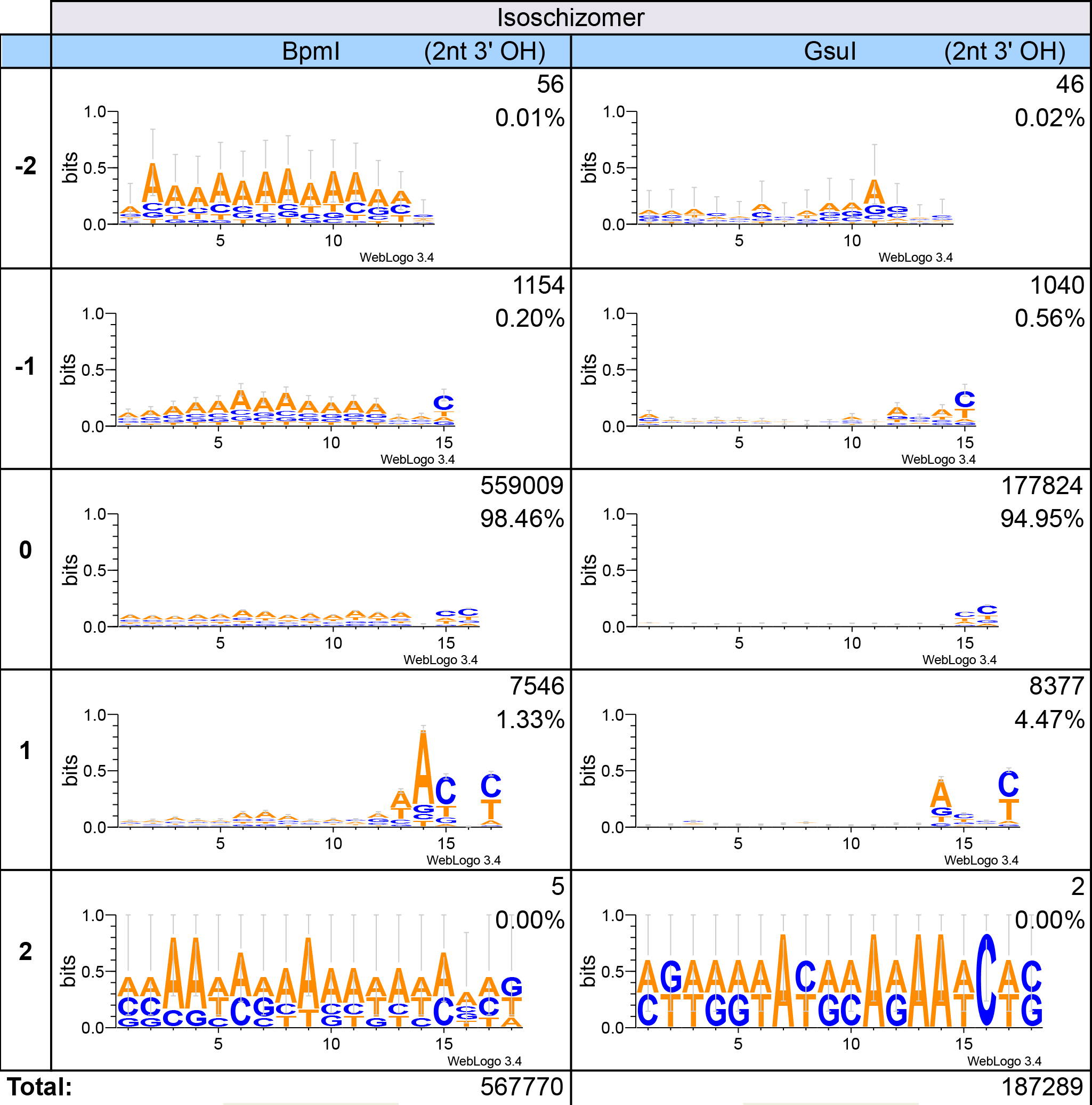

Supplement: S3 Fig — In grey is (in order from top) the type of overhang produced, number of sequences detected for that length and percent of total sequences detected within +/− 2 bp. Few sequences were detected at 2 bp distance, which make the sequence logos uncertain for those lengths. (TIF) [file pone.0117059.s005.tif]

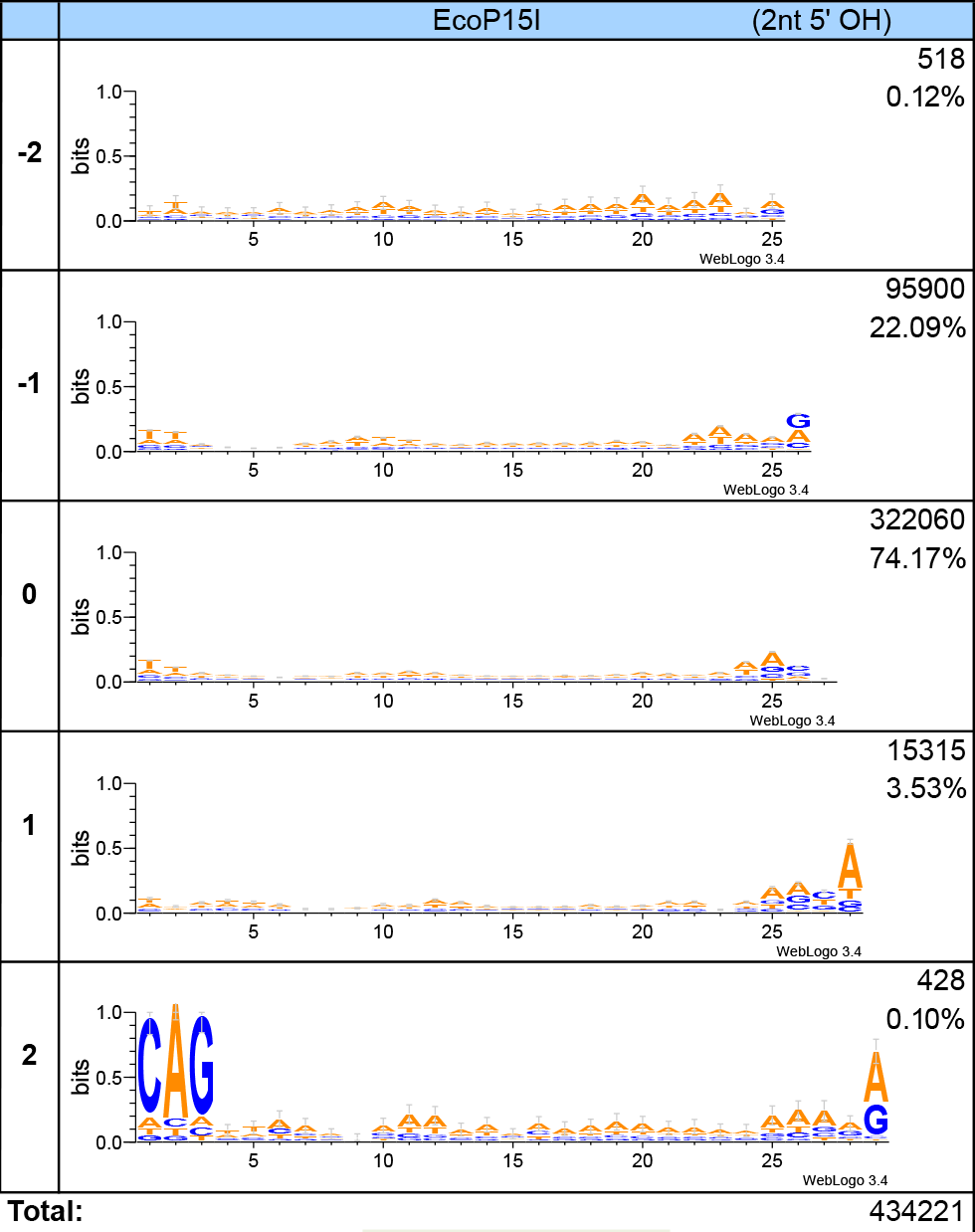

Supplement: S4 Fig — Two substrates were used to assay differences in A and G bases in the recognition sequence. In grey is (in order from top) the type of overhang produced, number of sequences detected for that length and percent of total sequences detected within +/− 2 bp. Few sequences were detected at 2 bp distance, which make the sequence logos uncertain for those lengths. (TIF) [file pone.0117059.s006.tif]

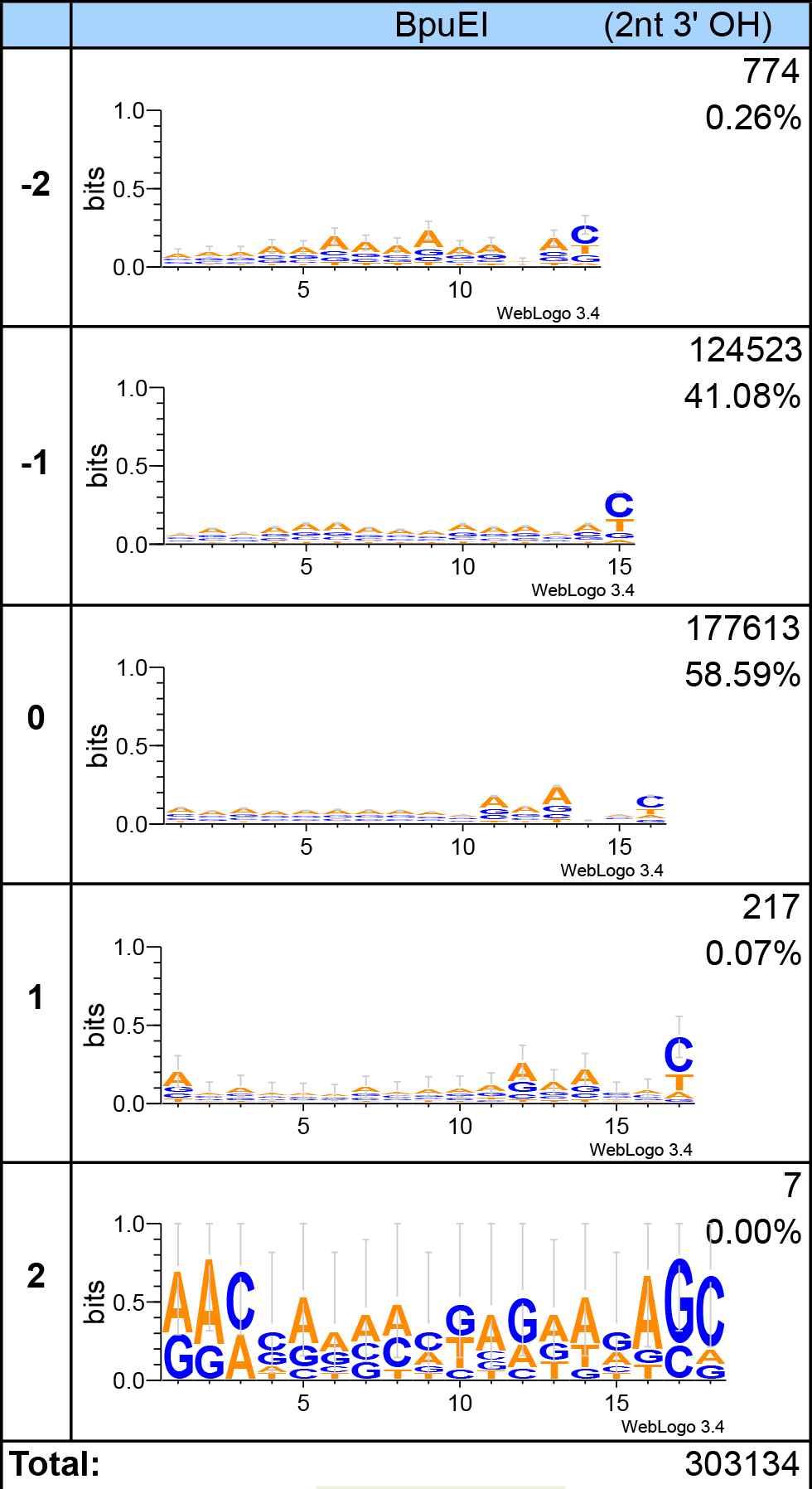

Supplement: S5 Fig — In grey is (in order from top) the type of overhang produced, number of sequences detected for that length and percent of total sequences detected within +/− 2 bp. Few sequences were detected at 2 bp distance, which make the sequence logos uncertain for those lengths. For this enzyme, a conserved motif “CAG” immediately proximal to the recognition sequence (CAGCAG) means the +2 detected here is actually a −1 slippage of a recognition sequence shifted three bases downstream. (TIF) [file pone.0117059.s007.tif]

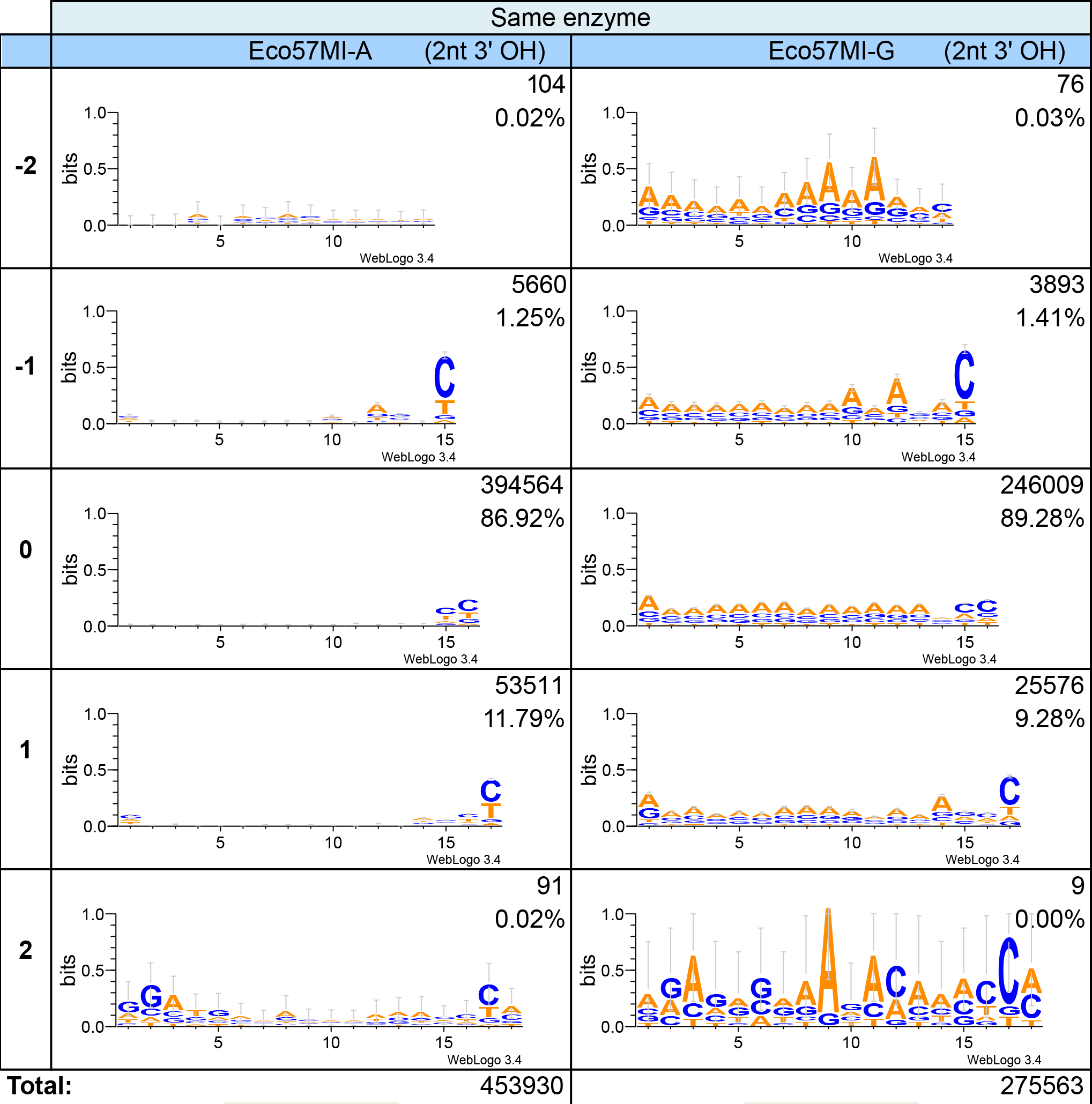

Supplement: S6 Fig — In grey is (in order from top) the type of overhang produced, number of sequences detected for that length and percent of total sequences detected within +/− 2 bp. Few sequences were detected at 2 bp distance, which make the sequence logos uncertain for those lengths. (TIF) [file pone.0117059.s008.tif]

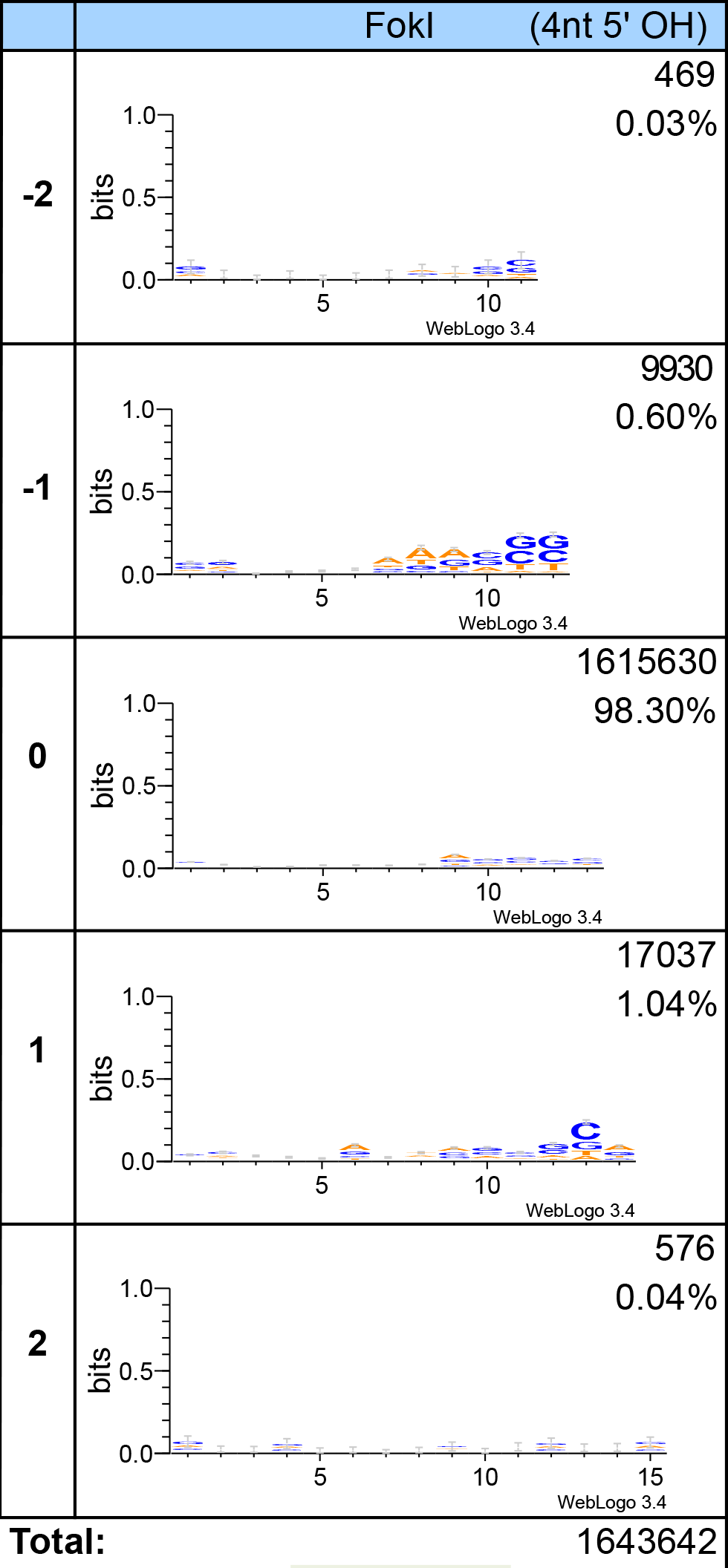

Supplement: S7 Fig — In grey is (in order from top) the type of overhang produced, number of sequences detected for that length and percent of total sequences detected within +/− 2 bp. Few sequences were detected at 2 bp distance, which make the sequence logos uncertain for those lengths. (TIF) [file pone.0117059.s009.tif]

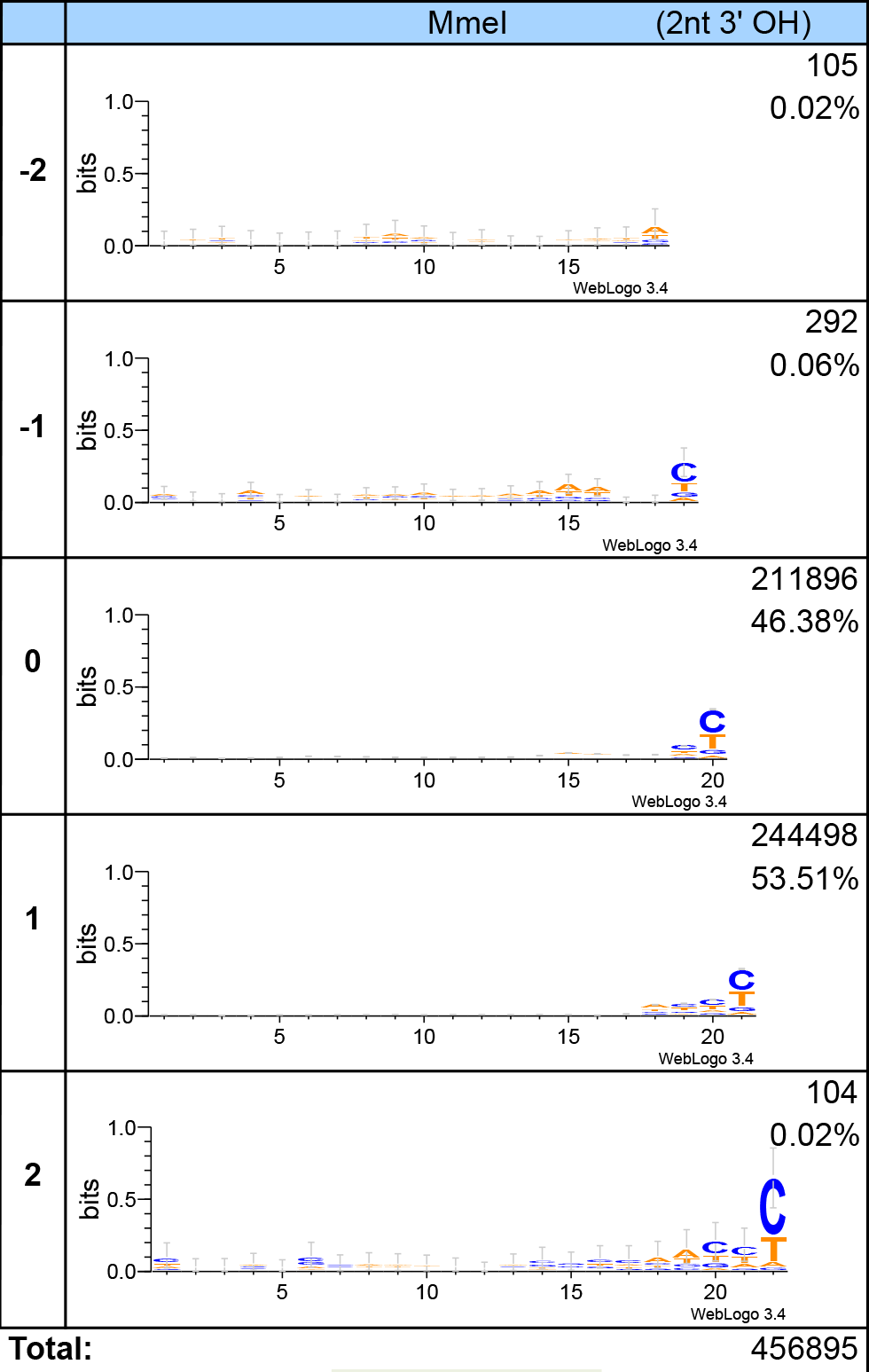

Supplement: S8 Fig — In grey is (in order from top) the type of overhang produced, number of sequences detected for that length and percent of total sequences detected within +/− 2 bp. Few sequences were detected at 2 bp distance, which make the sequence logos uncertain for those lengths. (TIF) [file pone.0117059.s010.tif]

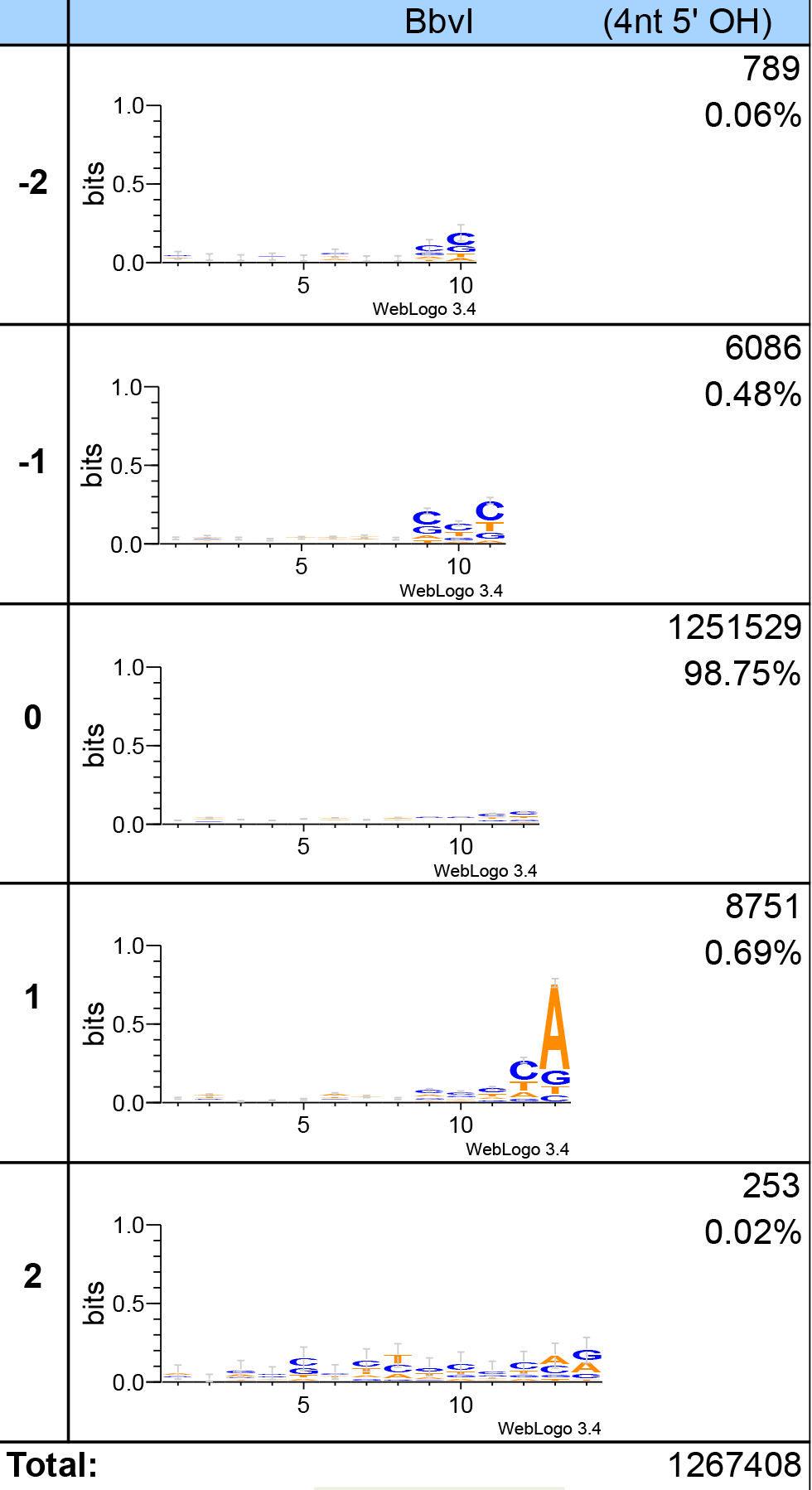

Supplement: S9 Fig — In grey is (in order from top) the type of overhang produced, number of sequences detected for that length and percent of total sequences detected within +/− 2 bp. Few sequences were detected at 2 bp distance, which make the sequence logos uncertain for those lengths. (TIF) [file pone.0117059.s011.tif]

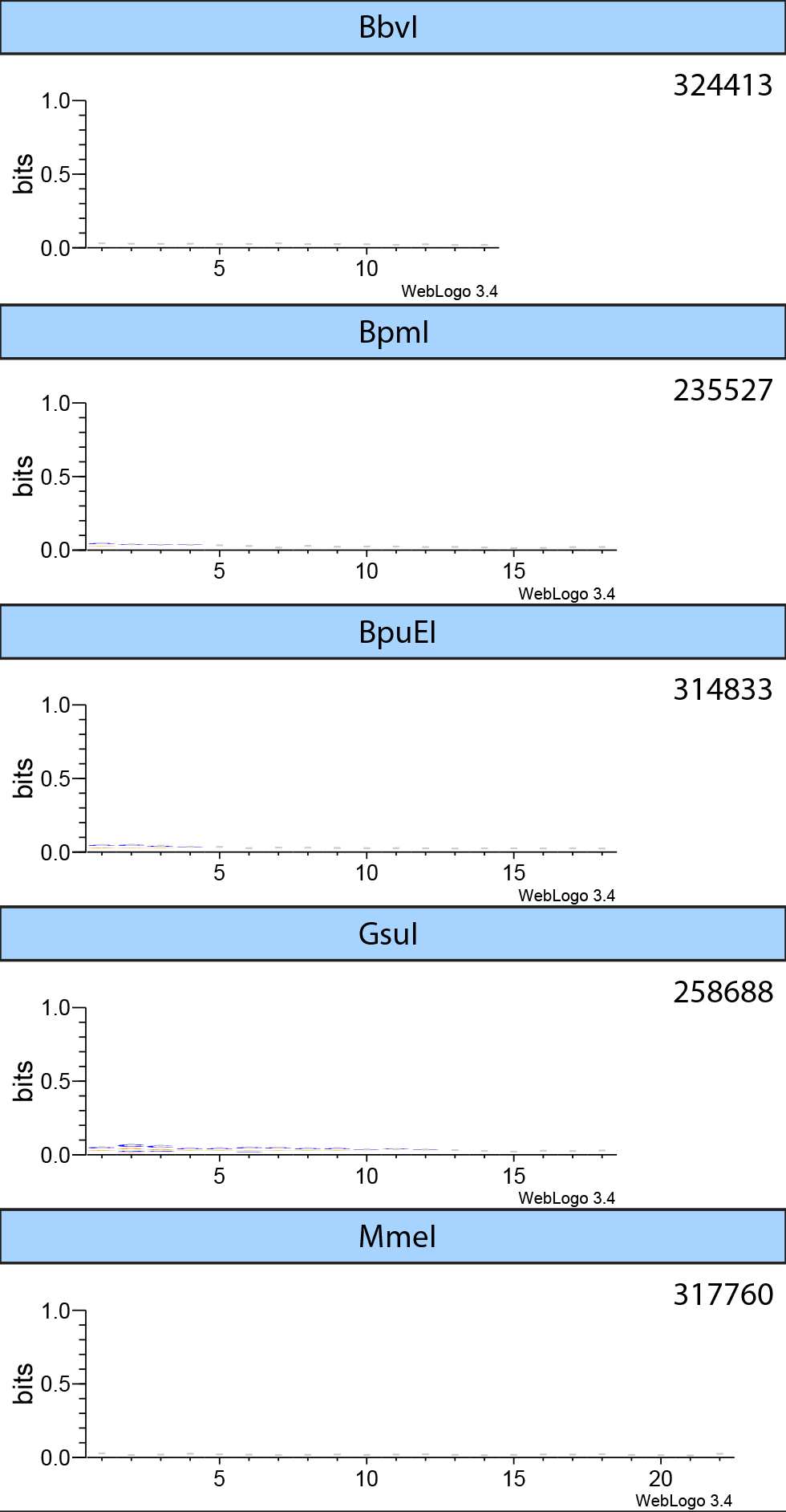

Supplement: S10 Fig — (TIF) [file pone.0117059.s012.tif]

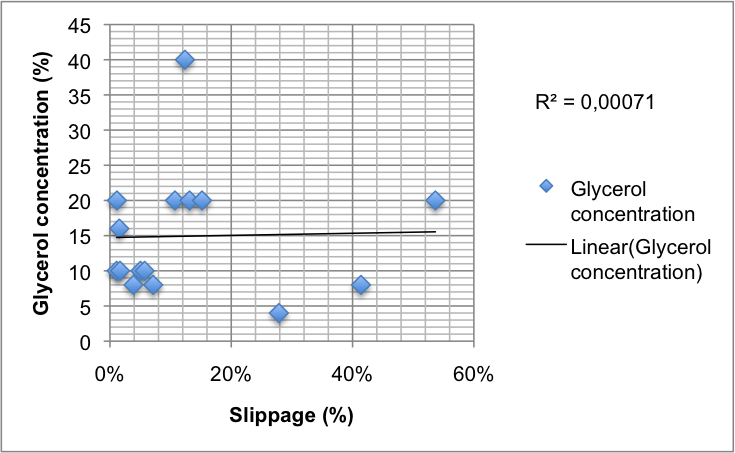

Supplement: S11 Fig — (PNG) [file pone.0117059.s013.png]

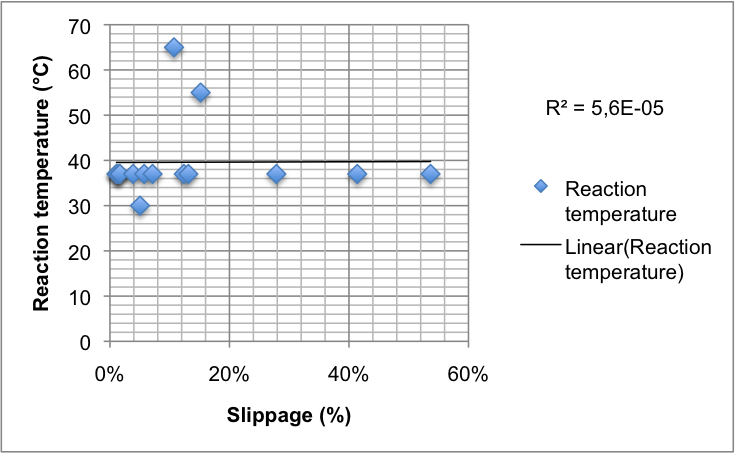

Supplement: S12 Fig — (PNG) [file pone.0117059.s014.png]

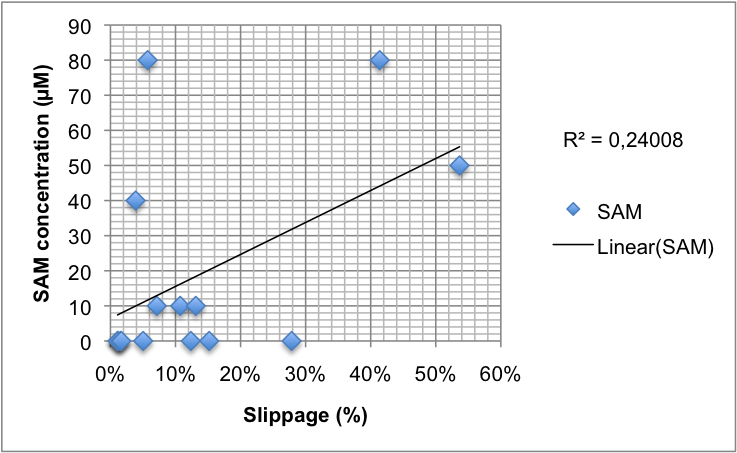

Supplement: S13 Fig — (PNG) [file pone.0117059.s015.png]

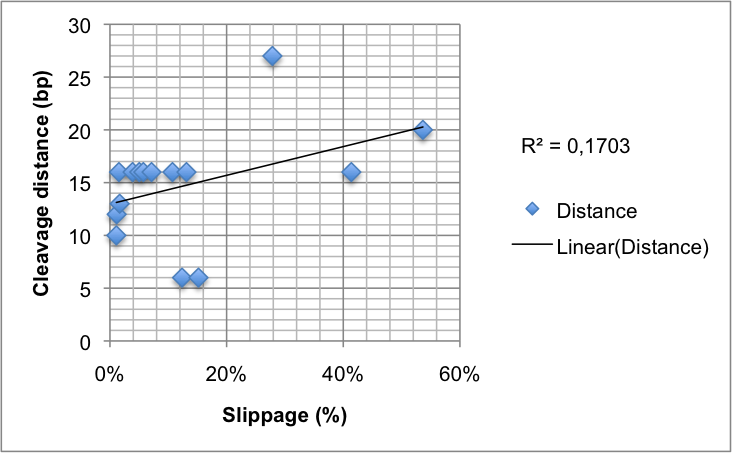

Supplement: S14 Fig — (PNG) [file pone.0117059.s016.png]

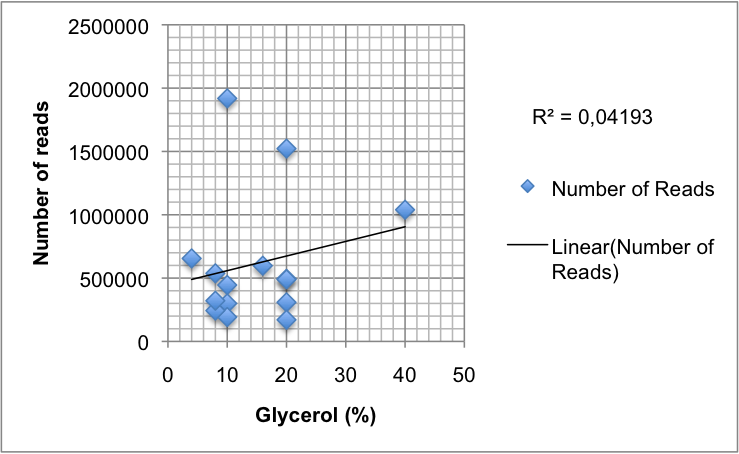

Supplement: S15 Fig — (PNG) [file pone.0117059.s017.png]

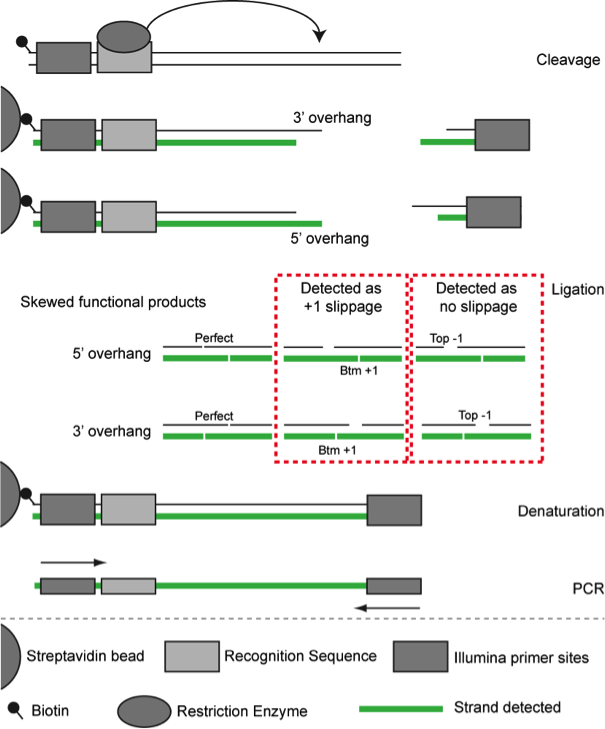

Supplement: S16 Fig — Although our model system was not designed to detect skewed slippage, two kinds of skewed product will give product. Since only the bottom strand is used for detection, bottom slippage of +1 and top slippage of −1 can form productive constructs, as indicated in the red box. Plus 1 bottom slippage is detected as +1 slippage, and −1 top slippage is detected as no slippage (false negative). These products would be disfavoured during ligation due to the gapped position and we expect these events, if they exist, to be low. (PNG) [file pone.0117059.s018.png]
