# Supplementary material for: Endonuclease Specificity and Sequence Dependence of Type IIS Restriction Enzymes
Source: PLoS One. 2015 Jan 28;10(1):e0117059. doi: 10.1371/journal.pone.0117059 (PMC4309577; doi:10.1371/journal.pone.0117059)
Supplement: S1 Table — (DOCX) [file pone.0117059.s019.docx]

**Table S1. Total slippage detected for all enzymes assayed.**

|  | **Reads detected between -2 and +2 slippage** | | | | | **Percent slippage** | | | | | **Total Slippage** |
| --- | --- | --- | --- | --- | --- | --- | --- | --- | --- | --- | --- |
|  | *-2* | *-1* | *0* | *1* | *2* | *-2* | *-1* | *0* | *1* | *2* |  |
| BseRI | 133 | 277 | 204168 | 1876 | 85 | 0.1% | 0.1% | 98.9% | 0.9% | 0.0% | 1.1% |
| AcuI | 122 | 3730 | 483042 | 1528 | 10 | 0.0% | 0.8% | 98.9% | 0.3% | 0.0% | 1.1% |
| BbvI | 789 | 6086 | 1251529 | 8751 | 253 | 0.1% | 0.5% | 98.7% | 0.7% | 0.0% | 1.3% |
| BpmI | 56 | 1154 | 559009 | 7546 | 5 | 0.0% | 0.2% | 98.5% | 1.3% | 0.0% | 1.5% |
| FokI | 469 | 9930 | 1615630 | 17037 | 576 | 0.0% | 0.6% | 98.3% | 1.0% | 0.0% | 1.7% |
| GsuI | 46 | 1040 | 177824 | 8377 | 2 | 0.0% | 0.6% | 94.9% | 4.5% | 0.0% | 5.1% |
| BsgI | 86 | 1434 | 391361 | 22282 | 7 | 0.0% | 0.3% | 94.3% | 5.4% | 0.0% | 5.7% |
| Eco57I | 31 | 2134 | 211086 | 14134 | 8 | 0.0% | 0.9% | 92.8% | 6.2% | 0.0% | 7.2% |
| Eco57MI-g | 76 | 3893 | 246009 | 25576 | 9 | 0.0% | 1.4% | 89.3% | 9.3% | 0.0% | 10.7% |
| SmuI | 521 | 1303 | 911072 | 125822 | 872 | 0.1% | 0.1% | 87.6% | 12.1% | 0.1% | 12.4% |
| Eco57MI-a | 104 | 5660 | 394564 | 53511 | 91 | 0.0% | 1.2% | 86.9% | 11.8% | 0.0% | 13.1% |
| FauI | 402 | 349 | 144788 | 24626 | 528 | 0.2% | 0.2% | 84.8% | 14.4% | 0.3% | 15.2% |
| EcoP15It2t | 518 | 95900 | 322060 | 15315 | 428 | 0.1% | 22.1% | 74.2% | 3.5% | 0.1% | 25.8% |
| BpuEI | 774 | 124523 | 177613 | 217 | 7 | 0.3% | 41.1% | 58.6% | 0.1% | 0.0% | 41.4% |
| MmeI | 105 | 292 | 211896 | 244498 | 104 | 0.0% | 0.1% | 46.4% | 53.5% | 0.0% | 53.6% |
