# Supplementary material for: Endonuclease Specificity and Sequence Dependence of Type IIS Restriction Enzymes
Source: PLoS One. 2015 Jan 28;10(1):e0117059. doi: 10.1371/journal.pone.0117059 (PMC4309577; doi:10.1371/journal.pone.0117059)
Supplement: S3 Table — The effects of storage conditions for each enzyme is shown added to 1x of the recommended reaction buffers. The final reaction conditions (not including supplements) depending on the amount of enzyme used for each reaction is shown. (DOCX) [file pone.0117059.s021.docx]

**Table S3. Enzyme storage and reaction buffer additions for the additional dataset.**

| **GsuI** | **BpuEI** | **MmeI** | **BpmI** |
| --- | --- | --- | --- |
| 10 mM Tris | 2.2 mM Tris | 2.2 mM Tris | 5.2 mM Tris |
| - | 6 mM Na+ | 6 mM Na+ | 14 mM Na+ |
| 0.2 mM K+ | 5 mM K+ | 5 mM K+ | - |
| 10 mM Mg2+ | 1 mM Mg2+ | 1 mM Mg2+ | 1 mM Mg2+ |
| 0.02 mM EDTA | 0.002 mM EDTA | 0.002 mM EDTA | 0.002 mM EDTA |
| 0.12 mM DTT | 0.12 mM DTT | 0.12 mM DTT | 0.12 mM DTT |
| 104 µg/ml BSA | 10 µg/ml BSA | 10 µg/ml BSA | 4 µg/ml BSA |
| 1% glycerol | 1 % glycerol | 1% glycerol | 1% glycerol |

*Note that this table only covers additions of enzyme and buffer, additional supplements such as BSA, ATP and SAM are not included, see Table 1 for those additions.
