# Supplementary material for: Endonuclease Specificity and Sequence Dependence of Type IIS Restriction Enzymes
Source: PLoS One. 2015 Jan 28;10(1):e0117059. doi: 10.1371/journal.pone.0117059 (PMC4309577; doi:10.1371/journal.pone.0117059)
Supplement: S4 Table — (DOCX) [file pone.0117059.s022.docx]

**Table S4. The different reaction conditions for each enzyme in the additional dataset**

| **Enzyme** | **Supplier** | **Temperature *(°C)*** | **Buffer** | **SAM *(µM)*** | **Enzyme amount *(U)*** | **Substrate amount *(pmol)*** | **Reaction Volume *(µl)*** | **Heat inactivation *(°C/min)*** |
| --- | --- | --- | --- | --- | --- | --- | --- | --- |
| BbvI | N | 37 | 4 | - | 2 | 20 | 50 | 65/20 |
| BpmI* | N | 37 | 2 | - | 2 | 20 | 50 | 65/20 |
| BpuEI | N | 37 | 3 | 80 | 2 | 20 | 50 | 65/20 |
| GsuI | T | 30 | B | - | 5 | 20 | 50 | 65/20 |
| MmeI | N | 37 | 4 | 50 | 2 | 20 | 50 | 80/20 |

*supplemented with BSA, 100 ug/ml

N=NEB

T=Thermo Scientific

SAM=S-adenosylmethionine
