# Supplementary material for: Endonuclease Specificity and Sequence Dependence of Type IIS Restriction Enzymes
Source: PLoS One. 2015 Jan 28;10(1):e0117059. doi: 10.1371/journal.pone.0117059 (PMC4309577; doi:10.1371/journal.pone.0117059)
Supplement: S5 Table — The additional dataset was analysed for slippage events within +/− 2 bp from the expected distance. To assess the impact of sequence errors a quality dataset (ca. 1.1 million 45 bp reads where all bases had quality scores above 20. i.e. less than 1% chance of sequencing error) was selected. (DOCX) [file pone.0117059.s023.docx]

**Table S5. Total slippage detected for all enzymes in the additional dataset.**

|  | **Unique reads detected between -2 and +2 slippage** | | | | | **Percentage slippage** | | | | | **Total slippage** | **Quality filtered** |
| --- | --- | --- | --- | --- | --- | --- | --- | --- | --- | --- | --- | --- |
|  | -2 | -1 | 0 | 1 | 2 | -2 | -1 | 0 | 1 | 2 |  |  |
| BbvI | 474 | 3856 | 487937 | 2602 | 34 | 0.1% | 0.8% | 98.6% | 0.5% | 0.0% | 1.4% | 1.4% |
| BpmI | 2 | 23 | 6301 | 208 | 0 | 0.0% | 0.4% | 96.4% | 3.2% | 0.0% | 3.6% | 3.5% |
| GsuI | 4 | 13 | 6522 | 505 | 2 | 0.1% | 0.2% | 92.6% | 7.2% | 0.0% | 7.4% | 7.3% |
| BpuEI | 252 | 118069 | 223398 | 197 | 8 | 0.1% | 34.5% | 65.3% | 0.1% | 0.0% | 34.7% | 34.6% |
| MmeI | 584 | 654 | 178364 | 176134 | 186 | 0.2% | 0.2% | 50.1% | 49.5% | 0.1% | 49.9% | 49.9% |
