# Supplementary material for: Endonuclease Specificity and Sequence Dependence of Type IIS Restriction Enzymes
Source: PLoS One. 2015 Jan 28;10(1):e0117059. doi: 10.1371/journal.pone.0117059 (PMC4309577; doi:10.1371/journal.pone.0117059)
Supplement: S6 Table — The dataset was analysed in three different ways to assay robustness. The “expected distance” was used as the primary analysis as it was judged to be most sensitive. “Entire read” analysis is based on perfect matches of a 10 bp recognition sequence right before the randomized position. No consideration was taken to at what distance the 10 bp matched. “Quality filtered” used on a limited high quality dataset (ca. 2 million 45 bp reads where all bases had quality scores above 20, i.e. less than 1% chance of sequencing error). Expected distance assumed the recognition sequence to be +/− 2 bp away from the recognition sequence and searched in order from longest to shortest distance for each read with a perfect match to a unique sequence for each enzyme. The results are similar and does not alter the general pattern identified, but can be viewed as an indication of the level of the sequencing assay induced uncertainty. (DOCX) [file pone.0117059.s024.docx]

**Table S6. The total amount of slippage detected +/- 2 bp away from the recognition sequence.**

|  | **Entire read** | **Quality filtered** | **Expected distance** |
| --- | --- | --- | --- |
| BseRI | 0.73% | 0.82% | 1.1% |
| BbvI | 1.10% | 1.06% | 1.3% |
| BpmI | 1.53% | 1.31% | 1.5% |
| FokI | 1.63% | 1.58% | 1.7% |
| AcuI | 3.86% | 3.35% | 1.1% |
| GsuI | 5.05% | 4.19% | 5.1% |
| BsgI | 5.79% | 5.10% | 5.7% |
| Eco57I | 7.06% | 6.03% | 7.2% |
| Eco57MI(G) | 10.72% | 9.08% | 10.7% |
| SmuI | 12.32% | 11.27% | 12.4% |
| Eco57MI(A) | 13.08% | 12.45% | 13.1% |
| FauI | 15.13% | 14.60% | 15.2% |
| EcoP15I | 26.53% | 24.63% | 25.8% |
| BpuEI | 41.45% | 39.04% | 41.4% |
| MmeI | 53.77% | 52.15% | 53.6% |
